# RBM10 deficiency promotes brain metastasis by modulating sphingolipid metabolism in a BBB model of *EGFR* mutant lung adenocarcinoma

**DOI:** 10.1186/s13046-025-03347-1

**Published:** 2025-03-11

**Authors:** Gang Xu, Bo An, Ruqiong Wang, Bo Pan, Huiting Hao, Xingmei Ren, Zihan Jing, Weitong Gao, Yajie Li, Yan Jin, Enguang Lin, Lihua Shang, Dexin Jia, Yan Yu

**Affiliations:** 1 Department of Medical Oncology, Harbin Medical University Cancer Hospital, Harbin, 150040 China; 2 Department of Clinical Laboratory, Harbin Medical University Cancer Hospital, Harbin, 150040 China; 3 Department of Medical Genetics, Harbin Medical University, Harbin, 150081 China; 4 Department of Pathology, Beidahuang Industry Group General Hospital, Harbin, 150081 China

**Keywords:** Brain metastasis, RBM10, Lung adenocarcinoma, Sphingolipid metabolism, BBB

## Abstract

**Background:**

Brain metastasis significantly contributes to the failure of targeted therapy in patients with epidermal growth factor receptor (EGFR)-mutated lung adenocarcinoma (LUAD). Reduced expression of RNA-binding motif protein 10 (RBM10) is associated with brain metastasis in these patients. However, the mechanism by which *RBM10* affects brain metastasis in EGFR-mutated LUAD remains unclear.

**Methods:**

An in vitro blood-brain barrier (BBB) model and brain metastasis-prone cell lines (BrM3) were established to confirm the brain metastatic potential of tumor cells following RBM10 knockdown. The roles of RBM10 and galactosylceramidase (GALC) in LUAD brain metastases were analyzed using cellular phenotypic assays and molecular biology techniques, including the combined analysis of Nanopore sequencing and CLIP-seq, minigene assays, and others.

**Results:**

This study demonstrates that RBM10 plays a vital role in inhibiting brain metastasis from EGFR-mutated LUAD by modulating sphingolipid metabolism. When RBM10 expression is low, GALC enters the nucleus to function. RBM10 deficiency inhibits exon skipping during GALC splicing, leading to upregulated GALC expression and increased sphingosine 1-phosphate (S1P) synthesis. S1P enhances BBB permeability, thereby promoting brain metastasis. Additionally, animal experiments show that the targeted agents Fingolimod (an S1P inhibitor) and RU-SKI-43 (a potential drug for RBM10 mutation) suppress the growth of brain metastasis.

**Conclusion:**

This study offers insights into the potential mechanisms of brain metastasis in LUAD and suggests a possible therapeutic target for further investigation.

**Supplementary Information:**

The online version contains supplementary material available at 10.1186/s13046-025-03347-1.

## Introduction

Brain metastasis from lung cancer is a major cause of treatment failure and mortality in patients with lung cancer [1, 2]. Epidermal growth factor receptor (*EGFR*) mutations are present in 66.3% of Asian non-smokers with lung adenocarcinoma (LUAD) [3], and brain metastasis is detected in up to 29% of these patients at initial diagnosis [4]. A clinical trial demonstrated that combination therapy with gefitinib and radiotherapy resulted in a 50% recurrence rate of brain metastasis in patients with *EGFR* mutations [5]. Moreover, the incidence of brain metastasis has been shown to be significantly higher in patients with *EGFR* mutations than in those with *EGFR* wild-type, as well as higher in patients with exon 19 deletion mutations (*19Del*) than in those with exon 21 mutations in *EGFR* [2]. Following a 3-year course of tyrosine kinase inhibitor (TKI) treatment in patients with lung cancer, the prevalence of brain metastasis escalated from 25% at initial diagnosis to 45% [6]. Brain metastasis in lung cancer has become an important reason for the failure of targeted therapy in patients with *EGFR* mutations. Therefore, there is a need to understand the mechanisms underlying brain metastasis in LUAD with *EGFR* mutations to develop effective prevention and treatment strategies for this condition.

Recent research has highlighted that brain metastasis of breast cancer is associated with lipid metabolism [7]. Furthermore, Jin et al. published a seminal article elucidating the pivotal role of brain lipid metabolism in mediating the metastasis of breast cancer cells to the brain [8]. Thus, the disruption of the blood-brain barrier (BBB) is closely related to lipid metabolism. Sphingosine (SPH), produced under the catalysis of galactosylceramidase (GALC) [9], can be converted to sphingosine 1-phosphate (S1P), a known promoter of tumor progression, under the action of sphingosine kinase 1/2 (SK1/2) [10]. S1P signaling is a key determinant of BBB permeability [11–13]. However, the relationship between brain metastasis from lung cancer and lipid metabolism has been largely unexplored, and the specific mechanism is not fully elucidated.

RNA binding motif protein 10 (RBM10) is a tumor suppressor involved in alternative splicing [14]. RBM10 exhibits a high mutation rate in various cancers, including LUAD, colon cancer, and liver cancer [15, 16]. Notably, in the Chinese population, *RBM10* mutations are present in about 8% of all LUAD cases, making it the fourth most common mutated gene in this cancer type [17]. These mutations lead to reduced RBM10 expression in LUAD, and this decrease is significantly higher in patients with stage IV LUAD than in those with stage I–III LUAD, suggesting a role for RBM10 in tumor metastasis [18, 19].

Our previous study revealed that RBM10 inhibits LUAD progression by regulating the Wnt/β-catenin signaling pathway, and RBM10 deficiency is associated with lung cancer metastasis, especially brain metastasis [20]. Building on this foundation, we hypothesize that RBM10 influences BBB permeability in *EGFR-*mutated lung cancer by regulating sphingolipid metabolism. Utilising a static 3D in vitro BBB model and the BM-prone cell lines (PC9BrM3 and 3255BrM3), we demonstrated increased brain metastatic potential upon RBM10 knockdown. We found that RBM10 regulated GALC in the sphingolipid pathway and elucidated how RBM10-mediated alternative splicing of GALC significantly impacts S1P synthesis. Specifically, RBM10 deficiency increases S1P production, thereby promoting brain metastasis.

## Experimental section

### Cell culture

Human LUAD cell lines (PC-9, H3255, H292), endothelial cells (HCMEC/D3), astrocytes (SVG), and pericytes (HBVP) were all obtained from the American Type Culture Collection (ATCC). All culture media were supplemented with 10% fetal bovine serum (FBS, PAN, Biotech GmbH, Germany) and antibiotics. H3255 and H292 cell lines were cultured in RPMI-1640 (Gibco^®^), while the remaining cell lines were cultured in Dulbecco’s Modified Eagle’s Medium (DMEM, Gibco^®^, Grand Island, NY, USA) at 37 °C in a humidified atmosphere containing 5% CO_2_. Cells used in experiments were in good condition without mycoplasma or saccharomycetes contamination. PC9 and 3255 BrM3 cells were used between passages 3 and 8 to ensure consistency in tumorigenicity and metastatic potential. Other cell lines were used between passages 5 and 30.

### Cell transfection

All shRNAs for RBM10 were obtained from Genechem Co., Ltd. (Shanghai, China). The used shRNA sequences were RBM10 shRNA-1, GCATGACTATGACGACTCA; RBM10 shRNA-2, CGACGGACATAAGGAGACA. A negative shRNA control (sh-NC) with the sequence 5’-UUCUCCGAACGUGUCACGUTT-3’ was also used. The used GALC siRNA sequences were Forward 5’-CAAGGUGGUUGAUGUUAUATT-3’ and Reverse 5’-UAUAACAUCAACCACCUUGTT-3’. The used GALC-S siRNA sequences were: Forward 5’-UGAUUUAGCUGGAUGGAUUtt-3’, and Reverse 5’- AAUCCAUCCAGCUAAAUCAtt-3’. The used UPF1 siRNA sequences were Forward 5’-AGACAGUCCUGGAGUGCUATT-3’ and Reverse 5’-UAGCACUCCAGGACUGUCUTT-3’. The siRNA (50µM, Ribobio) against GALC was reverse transfected at a 10 µl jet-PRIME (Poly-plus Transfection, France) in PC9BrM3 cells. The design of the RBM10 overexpression sequence and the packaging of lentiviruses were undertaken by Genechem Co., Ltd. (Shanghai, China). The PC9BrM3 cell lines were infected with the lentivirus and subsequently selected with puromycin (2 µg/ml) for 14 days to establish stable cell lines. The transfection efficiencies were verified by western blot (WB).

### Construction of in vitro BBB models

Human brain microvascular endothelial cells, pericytes, and astrocytes were used to establish six distinct in vitro BBB models, classified according to cell type and spatial arrangement. The pericytes (2 × 10^4^ cells/cm^2^) or astrocytes (1 × 10^5^ cells/cm^2^) were seeded on the bottom side of the Matrigel-coated polyester membranes in Transwell inserts (Corning Life Sciences, USA). These cells were left to adhere firmly overnight, following which endothelial cells (2 × 10^5^ cells/cm^2^) were seeded on the inside or upper side of the inserts placed in the well of the 12-well culture plates devoid of cells. The cultures were then maintained for an additional 3 days. Subsequently, the transepithelial electrical resistance (TEER) was measured to assess barrier integrity, and the BBB was considered successfully constructed when the resistance readings stabilized.

### Trans-BBB migration assay

PC9BrM3 sh-NC and shRBM10 cells were labeled with 5 µM of CellTracker Green CFMDA (Shanghai Maokang Biotechnology Co., Ltd, China) in a serum-free medium for 45 min. Subsequently, these cells were inoculated into the upper chamber of the inserts at a density of 10,000 cells in 200 µl of DMEM medium containing 0% FBS. The lower chamber was filled with 600 µl of DMEM medium containing 10% FBS. Following overnight incubation, the inserts were removed, and the number of cells that had migrated to the lower chamber was recorded by a positive fluorescence microscope (Motic China Group Co., Ltd., China).

### Western blot

The standard western blot (WB) experiment was conducted as previously described [21], utilising 30 µg of protein samples extracted from cells. The antibodies employed for the WB analysis are listed in Table S1.

### Immunofluorescence staining

For cell immunofluorescence staining (IF), the cells were incubated overnight at 4 °C with rabbit polyclonal antibodies against Claudin-5, Zo-1, P-gp, and GFAP, as well as a mouse polyclonal antibody against CK-18 (Table S1). The following day, the corresponding secondary antibodies were applied to the cells and incubated for 1 h at room temperature. The nuclei were stained with DAPI for 5 min. The cells were then imaged using a positive fluorescence microscope.

### Quantitative real-time PCR (qRT-PCR) analysis

The total RNA was extracted from LUAD cells utilising TRIzol reagent (Invitrogen, Carlsbad, CA, USA) following the manufacturer’s protocol. Subsequently, cDNA synthesis was performed using the Fast Quant RT Kit (TIANGEN, China). GAPDH was employed as the internal control. Quantitative real-time PCR (qRT-PCR) analysis was conducted on a 7500 Fast PCR System (Applied Biosystems, Foster City, CA, USA) with Talent qPCR Pre-Mix (SYBR Green; TIANGEN, China). The relative mRNA expression levels were quantified using the 2 − ΔΔCT method. The primer sequences utilized for qRT-PCR are listed in Table S2.

### CLIPSeq

The CLIP-Seq experiment was collaboratively conducted by our research group and IEMed Guangzhou Biomedical Technology Co., Ltd (Guangzhou, China). Crosslinking-immunoprecipitation assays utilised the CLIP Kit (IEMed, K319) and the RBM10 antibody (HPA034972, Sigma). Detailed CLIP operation procedures are illustrated in [22]. Subsequently, high-throughput sequencing of RNA fragments was conducted, followed by comprehensive bioinformatics analyses aligned with RNA-Seq.

### Minigene splicing reporter assays

To investigate the splicing of target exons following RBM10 overexpression (OE) or knockdown (KD), we co-transfected minigene splicing reporters and shRNA oligonucleotides targeting the RBM10 coding sequence into PC9 or 3255 cells. For RBM10 OE, PC9 or 3255 cells were seeded into six-well plates (4 × 10⁵ cells per well), cultured for 24 h, and transfected with 500 ng of minigene reporter using jetMESSENGER^®^ (PolyPlus-transfection). For RBM10 KD, minigene plasmids were transfected into the cells 24 h after shRNA transfection using jetMESSENGER^®^ (PolyPlus-transfection). Cells were harvested 48 h post-shRNA transfection for total RNA extraction, cDNA synthesis, and PCR analysis.

### RNA Immunoprecipitation (RIP)

PC9BrM3 and 3255BrM3 cells were collected at the logarithmic growth phase and dissociated at 4 °C for 1 h. The prepared magnetic beads were incubated with the antibody mixture for 2 h, and the final product was purified. RIP was performed using a RIP Kit (*Geneseed*, China) according to the manufacturer’s instructions. The antibodies used were anti-RBM10 (HPA034972, Sigma, Germany) and a secondary IgG antibody.

### RNA pull-down assay

RNA pull-down was performed using Pierce™ Magnetic RNA–protein pull-down kit (Thermo, USA) according to its instructions. The RNA–protein complex was analysed by western blot.

### Chromatin immunoprecipitation (ChIP)

9003 SimpleChIP(R) Kit **(Cell Signaling Technology**, USA) was used for ChIP assay. ChIP grade anti-RNA polymerase II CTD repeat YSPTSPS antibody (ab252855, Abcam, Britain) was used to precipitate chromatin bound to the Pol II pSer2 protein. Subsequent qPCR was performed as described previously, and the results are expressed as a percentage of the input. Specific ChIP primers used for PCR were listed in Supplementary Table S2.

### Animal experiments

Female nude mice (BALB/c, 4 weeks) were procured from Beijing Vital Li Hua Experimental Animal Technology Company (Beijing, China). All animal procedures were conducted by the instructions of the Institutional Animal Care and Use Committee (IACUC) at the Second Affiliated Hospital of Harbin Medical University, China, and the NIH Guide for the Care and Use of Laboratory Animals. Tumor cells (PC9/3255) were labeled with luciferase-expressing lentiviral particles. To establish the BM model, 5 × 10^5^ tumor cells suspended in 100 µl of PBS were inoculated into the left cardiac ventricle. Brain metastatic cell populations (i.e.PC9-BrM1/3255-BrM1) were isolated through one round of in vivo selection following the initial isolation and in vitro culturing of PC9/3255 cells forming BM. Subsequently, BrM3 was established using the same approach.

Five-week-old female mice were anaesthetised and secured in a stereotactic frame, with the head held firmly in place by gentle pressure using ear bars whilst maintaining deep anaesthesia. A scalp incision of one centimetre was performed to expose the bregma, which was used as the origin for coordinate referencing (x = 0 mm, y = 0 mm, z = 0 mm). Subsequently, a burr hole was drilled in the skull on the right hemisphere at coordinates x = 1.5 mm, y = 1 mm, and z = 0 mm. Each animal received an injection of approximately 300,000 cells per 5 µl (z = 2 mm).

Osimertinib (an EGFR inhibitor) and Fingolimod (an S1P inhibitor) were procured from MedChemExpress. All agents were utilised by the manufacturer’s instructions. Beginning on day 7 post-procedure, the mice were treated with these agents via oral gavage, continuing 5 days per week for the remainder of the experiment. These dosages were 25 mg/kg for Osimertinib and 5 mg/kg for Fingolimod [23, 24].

In preparation for bioluminescence imaging, the mice received 10 µl/g of D-luciferin (15 mg/ml, Beijing Solarbio Science & Technology Co., Ltd., China) via intraperitoneal injection 10–15 min before the imaging session. Bioluminescence signals were then recorded using the AniView SE Imaging System (Guangzhou Biolight Biotechnology Co., Ltd., China), adhering strictly to the manufacturer’s protocol.

### Immunohistochemistry

Immunohistochemistry (IHC) was conducted following the method previously described.^(21)^ For the primary antibody incubation, RBM10 (HPA034972, Sigma,1:300) and GALC (11991-AP, Proteintech,1:150) were employed for IHC. Eventually, an Olympus microscope (Toyo, Japan) was utilised for the examination of the tissue sections.

### Statistical analysis

Each experiment was independently conducted 3 times. Statistical analysis was carried out using GraphPad Prism software, version 9.4.0 (San Diego, California, USA). Results were presented either as the mean ± standard deviation (SD) or the median, as appropriate. Data analyses involved the application of Student’s t-test for comparisons between two groups or one-way analysis of variance (ANOVA) for comparisons involving three or more groups. A P value less than 0.05 was considered indicative of statistical significance.

**Fig. 1:**
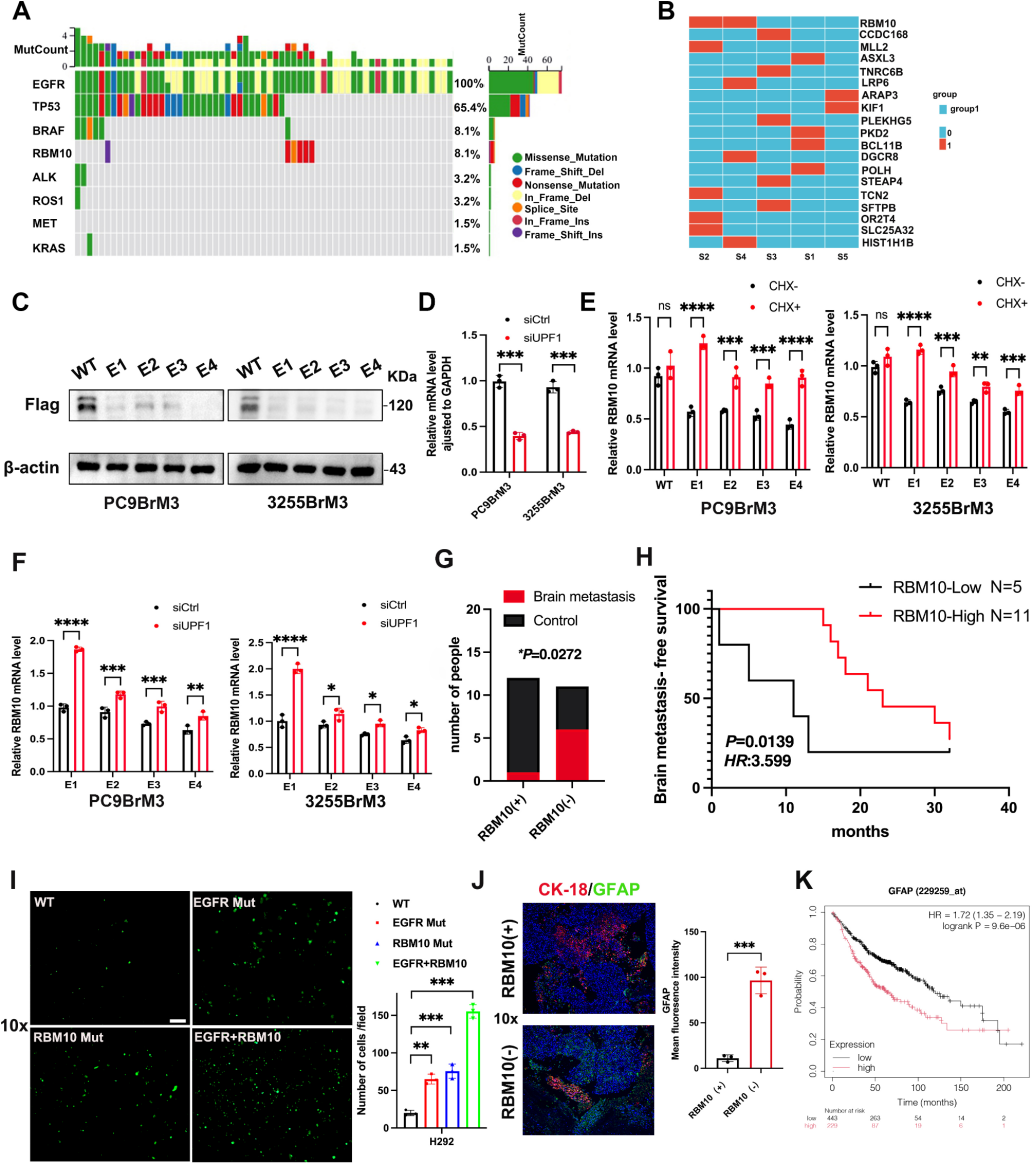
RBM10 mutations lead to decreased expression and are associated with lung cancer brain metastasis. (**A**) EGFR and RBM10 co-mutations in the TCGA database. (**B**) WES results of five lung adenocarcinoma and paracancerous samples. (**C**) Western blot (WB) was used to detect RBM10 expression after RBM10 mutations. (**D**) RT-qPCR was used to detect UPF1 expression after UPF1 knockdown. (**E**) RT-qPCR was used to detect RBM10 expression after CHX treatment. (**F**) RT-qPCR was used to detect RBM10 expression after siUPF1 treatment. (**G**) The expression of RBM10 is associated with brain metastasis. (**H**) Brain metastasis-free survival curves for patients with high and low RBM10 expression, respectively (*p* = 0.0139). (**I**) Trans-BBB migration assay was used to test the transfer capacity under different mutant conditions. (**J**) Immunofluorescence was performed on paraffin sections (Red: CK-18, Green:GFAP, Blue: DAPI). Scale bar: 10x:50 μm. (**K**) The Kaplan-Meier plotter database was searched for the overall survival of lung adenocarcinoma patients. *P < 0.05, **P < 0.01, ***P < 0.001

## Results

### Development of in vitro and in vivo BBB models

To study the mechanism of lung cancer brain metastasis, we developed in vitro and in vivo BBB models. Human brain microvascular endothelial cells, pericytes, and astrocytes were categorized into six distinct in vitro BBB models based on the cells type and the location of the cells (Fig. S1A). The results of the 4-hour liquid leakage assay indicate that Model 6 has the highest BBB integrity (Fig. S1B). Trypan blue was added to the upper chamber of the six models, and optical density (OD) values were recorded after a 30-minute incubation period. The OD value was found to be the lowest for Model 6 (Fig. S1C). Transendothelial electrical resistance (TEER) was measured across all six BBB models, with Model 6 demonstrating the highest TEER, which gradually increased over time (Fig. S1D). Immunofluorescence (IF) analysis demonstrated the strongest expression of Claudin-5 (green) and ZO-1 (red) in the microvascular endothelial cells of Model 6, with increasing levels over time (Fig. S1E). The expression of tight junction proteins between cells was evident at a 40x magnification in Model 6 (Fig. S1F). Additionally, Western blot analysis further confirmed that the expression of functional and structural markers, namely, glucose transporter protein-1 (GLUT-1), p-glycoprotein (P-gp), Claudin-5, and zonula occluden-1 (ZO-1), was highest in Model 6 among the six BBB models (Fig. S1G). Consequently, Model 6, featuring a three-cell full-contact co-culture configuration, was established as the optimal in vitro BBB model.

**Fig. 2:**
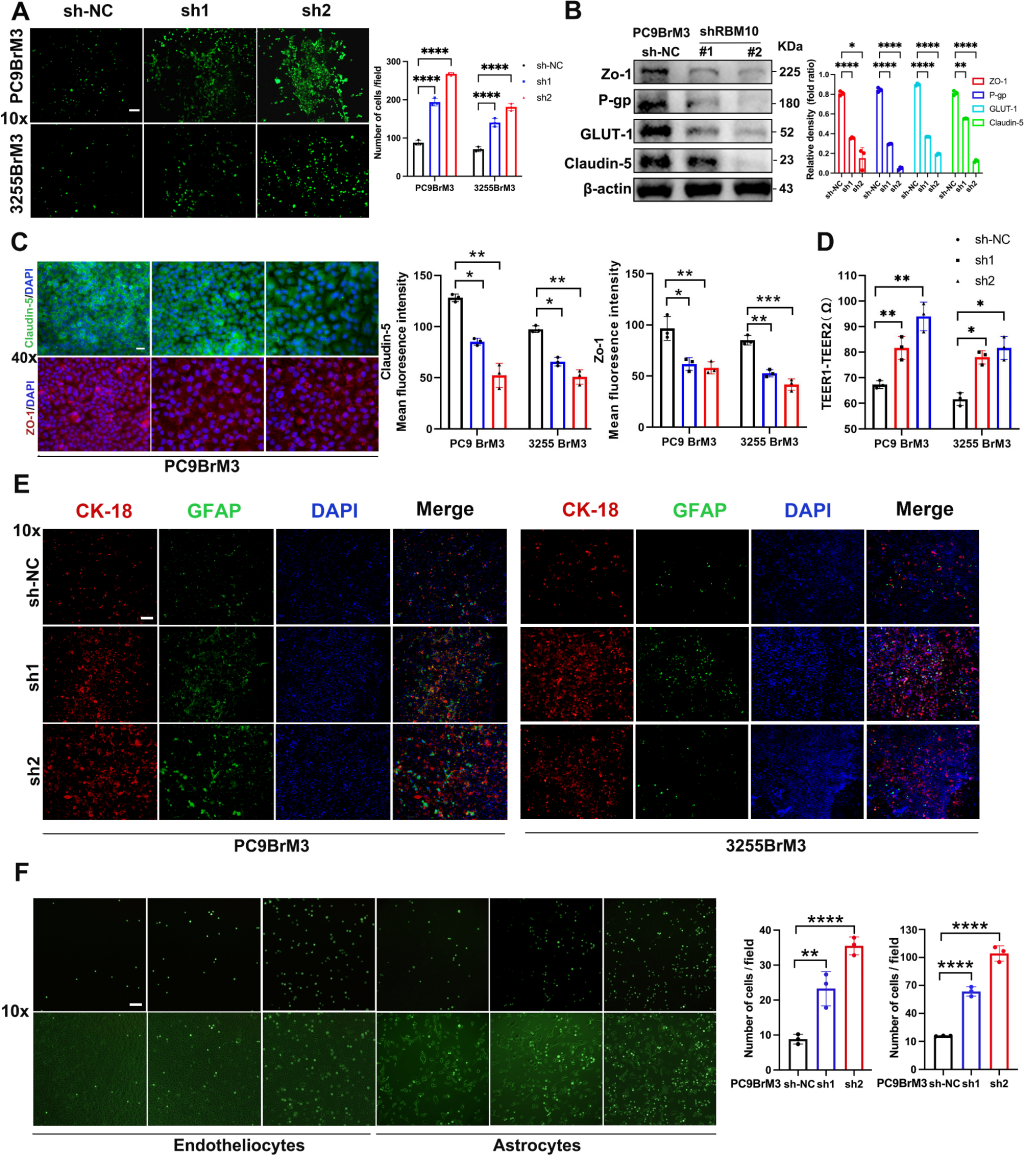
Knockdown of RBM10 promotes the migration of PC9BrM3 and 3255BrM3 cells in an in vitro BBB model. (**A**) Trans-BBB migration assay was used to test the transfer capacity of LUAD EGFR mutation cells with RBM10 knockdown. (**B-C**) Tumor cells were co-cultured with vascular endothelial cells. Endothelial cells were then collected for WB and IF. (**D**) TEER in the upper compartment of the BBB model was measured (TEER1 is the resistance measured after successful BBB construction; TEER2 is the resistance measured after the addition of tumor cells). (**E**) The number of tumor-associated astrocytes was observed (Red: CK-18, Green: GFAP). (**F**) Cell–Cell Adhesion Assay: The adhesion of tumor cells to vascular endothelial cells and astrocytes was observed. Scale bars: 10x:50 μm, 40x:20 μm. *P < 0.05, **P < 0.01, ***/****P < 0.001

The *EGFR 19Del*-harboring PC9 cell line was transfected with a fluorescent viral gene and injected into the left ventricle of nude mice. Imaging at 53 days post-injection revealed brain metastasis formation. At 73 days, the mice were sacrificed, and primary cells were extracted (Fig. S2A). The 3255 cell line with the EGFR L858R mutation was established using the same method. Two rounds of screening were performed in nude mice to generate PC9 and 3255 BrM3 cells with a propensity for brain metastases, followed by subsequent experiments using the BrM3 cell lines. The BrM3 cells were identified through morphological evaluation, fluorescent viral gene labeling, and specific tumor markers (Fig. 2B-E). Compared to their parental counterparts, the PC9BrM3 cell lines exhibit significant changes in proliferation, migration, and EMT, which may be linked to their increased metastatic potential (Figures S3A-E).

### Co-mutation of RBM10 and EGFR is associated with lung cancer brain metastasis

According to the FLAURA trial [25], osimertinib is the current standard first-line therapy for patients with metastatic NSCLC harboring *EGFR 19del* and *L858R* mutations. A phase II multicenter study also showed that tumor genomic mutations TP53 and RBM10 were the most frequently co-occurring with *EGFR* mutations [26], We found from The Cancer Genome Atlas (TCGA) that approximately 8.1% of *EGFR*-mutated LUAD patients harbor RBM10 mutations (Fig. 1A). We performed whole-exome sequencing (WES) on five LUAD primary tumor–normal pairs from patients who later developed brain metastasis (Fig. 1B). The frequency of RBM10 mutations was 40% (2/5). According to the cBioPortal database, RBM10 truncating mutations are predominant (Fig. S4A). In the TCGA-LUAD database, the RBM10 mutation leads to a reduction in RBM10 expression (Fig. S4B). In patients with stage Ib-IV disease in TCGA-LUAD, RBM10 mutations are associated with a worse prognosis (Fig. S4C). An analysis of data from the HCMDB database, which included 77 lung cancer patients, revealed that among 14 patients with lung adenocarcinoma, 6 developed brain metastases with reduced RBM10 expression (Fig. S4D).

We identified several frameshift mutations in RBM10 across different exons and transcripts (Table S3). We selected four of these mutations for functional studies to construct plasmids (Table S4). These mutations introduce premature termination codons (PTC), triggering nonsense-mediated mRNA decay (NMD), resulting in the decreased expression of RBM10, as observed by Western blot (Fig. 1C). Classical NMD, which is translation-dependent and involves core proteins like UPF1, was examined. First, we inhibited UPF1 expression with siRNA (Fig. 1D). To further verify whether classical translation-dependent NMD effects occurred with mutants E1, E2, E3, and E4 did occur, we treated PC9BrM3 and 3255BrM3 cells transfected with WT and mutant constructs with cycloheximide (CHX). The qRT-PCR analysis showed significant changes in mRNA levels (*P* < 0.05). These data further confirm that the mutants E1, E2, E3 and E4 triggered NMD, leading to mRNA degradation and inhibition of translation. When CHX was added to transfected cells, thereby blocking the effect of NMD [27], mRNA expression levels increased (Fig. 1E). E1, E2, E3 and E4 plasmids and UPF1-siRNA were co-transfected into PC9BrM3 and 3255BrM3 cells for 24 h, Using the GAPDH gene was used as an internal control, we found that, compared with control, the expression levels of RBM10 normal transcripts from E1, E2, E3 and E4 mutant plasmids were significantly increased after UPF1 knockdown (Fig. 1F). RBM10 mutations are distributed throughout the gene, but they mostly result in low expression. Therefore, to facilitate follow-up research, we focused on the low expression caused by RBM10 mutations. And then, IHC analyses were conducted on 13 paired samples of LUAD and corresponding brain metastasis. RBM10 expression was low in 11 cases and high in 2 cases (Fig. S4E). Although no significant difference in RBM10 expression was observed between primary lung lesions and brain metastases (Fig. S4G), RBM10 expression levels in the two sites were significantly correlated (Fig. S4H).

To further investigate the role of RBM10 in brain metastases, we collected clinicopathological data from 23 lung adenocarcinoma patients with EGFR mutations. Among them, 7 patients were diagnosed with brain metastases at initial presentation, while the remaining 16 patients were followed up to generate brain metastasis-free survival (BMFS) curves. IHC analysis showed that RBM10 expression was associated with brain metastasis in lung cancer patients with *EGFR* mutations; the low expression group had a higher rate of brain metastasis (Fig. 1G). Patients with high RBM10 expression demonstrated longer BMFS than those with low expression (23 months vs. 11 months, *p* = 0.0139; Fig. 1H). Experiments using CellTracker Green (CFMDA)-labeled cells suggested that co-mutation of EGFR and RBM10 could promote brain metastasis in lung cancer cells (Fig. 1I). Futhermore, the number of glial fibrillary acidic protein (GFAP)–positive astrocytes, a marker of brain metastasis, also increased, suggesting mutual stimulation between tumor cells and astrocytes. The interaction between astrocytes and cancer cells significantly impacts the treatment outcomes and prognosis of lung cancer patients with brain metastases, while also creating a microenvironment conducive to tumor growth [28, 29]. IF of paraffin sections of brain metastatic tumors in patients with LUAD further showed increased GFAP-positive astrocytes in the lesions of patients with low RBM10 expression (Fig. 1J). The Kaplan–Meier plotter (KMplotter) database revealed poorer prognosis in patients with LUAD exhibiting high *GFAP* expression(Fig. 1K).

### in vitro and in vivo BBB model verifies that RBM10 knockdown promotes brain metastasis in PC9BrM3 and 3255BrM3 cells

RBM10 knockdown increased the number of cells crossing the BBB (Fig. 2A) and decreased the expression of GLUT-1, P-gp, Claudin-5, and ZO-1 in vascular endothelial cells (Fig. 2B), leading to enlarged junctional gaps between vascular endothelial cells (Fig. 2C). To verify the role of RBM10 in cancer-astrocyte interactions, we cocultured PC9BrM3 and 3255BrM3 sh-NC, shRBM10-1, and shRBM10-2 cells with Model 6. After 48 h, IF revealed an increased number of tumor cells and GFAP-positive astrocytes and decreased TEER in the shRBM10 group (Fig. 2D-E). A cell adhesion assay showed an increased number of cells in the shRBM10 group, suggesting enhanced adhesion between tumor cells and both endothelial cells and astrocytes (Fig. 2F). Conversely, RBM10 overexpression reduced the number of tumor cells crossing the BBB in the in vitro BBB model (Fig. S5). Taken together, these findings demonstrate that RBM10 knockdown promotes the brain metastatic capacity of PC9BrM3 and 3255BrM3 cells in in vitro BBB models.

**Fig. 3:**
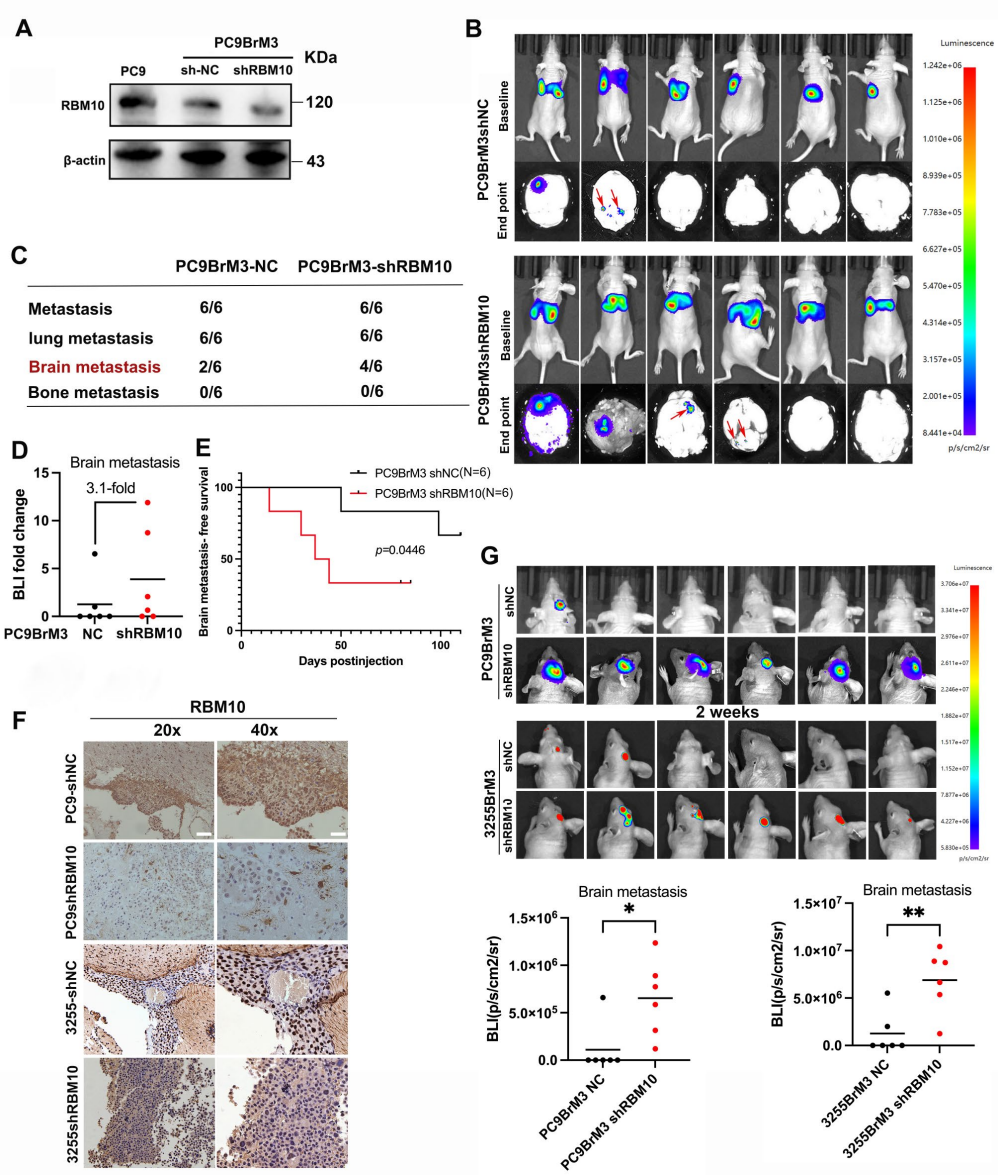
The brain metastatic potential of the PC9BrM3 and 32559BrM3 cells was validated using an in vivo model after RBM10 knockdown. (**A**) WB was used to detect RBM10 expression. (**B**) Bioluminescence imaging (BLI) in mice receiving intracardiac injection of wild-type (WT) or RBM10-knockdown PC9BrM3 cells. (**C**) Frequency and distribution of metastasis following inoculation of 5 × 10^5^ cells. (**D**) Quantification of relative fold change using BLI. (**E**) Brain metastasis-free survival curves for mice injected with wild-type (WT) and RBM10-knockdown cells, respectively (*p* = 0.0446). (**F**) Representative images of RBM10 IHC analysis of mouse brain metastasis samples. (**G**) The mice received an intracranial injection of wild-type (WT) or RBM10-knockdown PC9BrM3 and 3255BrM3 cells, and then BLI was performed after two weeks. Scale bars: 20x:40 μm, 40x:20 μm. *P < 0.05, **P < 0.01

To investigate the role of RBM10 in vivo, brain metastasis animal models were established using intracardiac injection and intracranial orthotopic injection methods. After RBM10 knockdown, the expression of RBM10 protein was reduced (Fig. 3A), and the incidence of brain metastases increased accordingly (Fig. 3B-C). The fluorescence intensity in the brain metastasis group was 3.1 times higher than that in the non-knockdown group (Fig. 3D). Additionally, the brain metastasis-free survival (BMFS) in the shRBM10 group was significantly shorter (Fig. 3E). The expression of RBM10 was confirmed by IHC, RBM10 is localized in the nucleus. (Fig. 3F). Intracranial injection further demonstrated that RBM10 knockdown significantly increased the incidence of intracranial tumors (Fig. 3G).

**Fig. 4:**
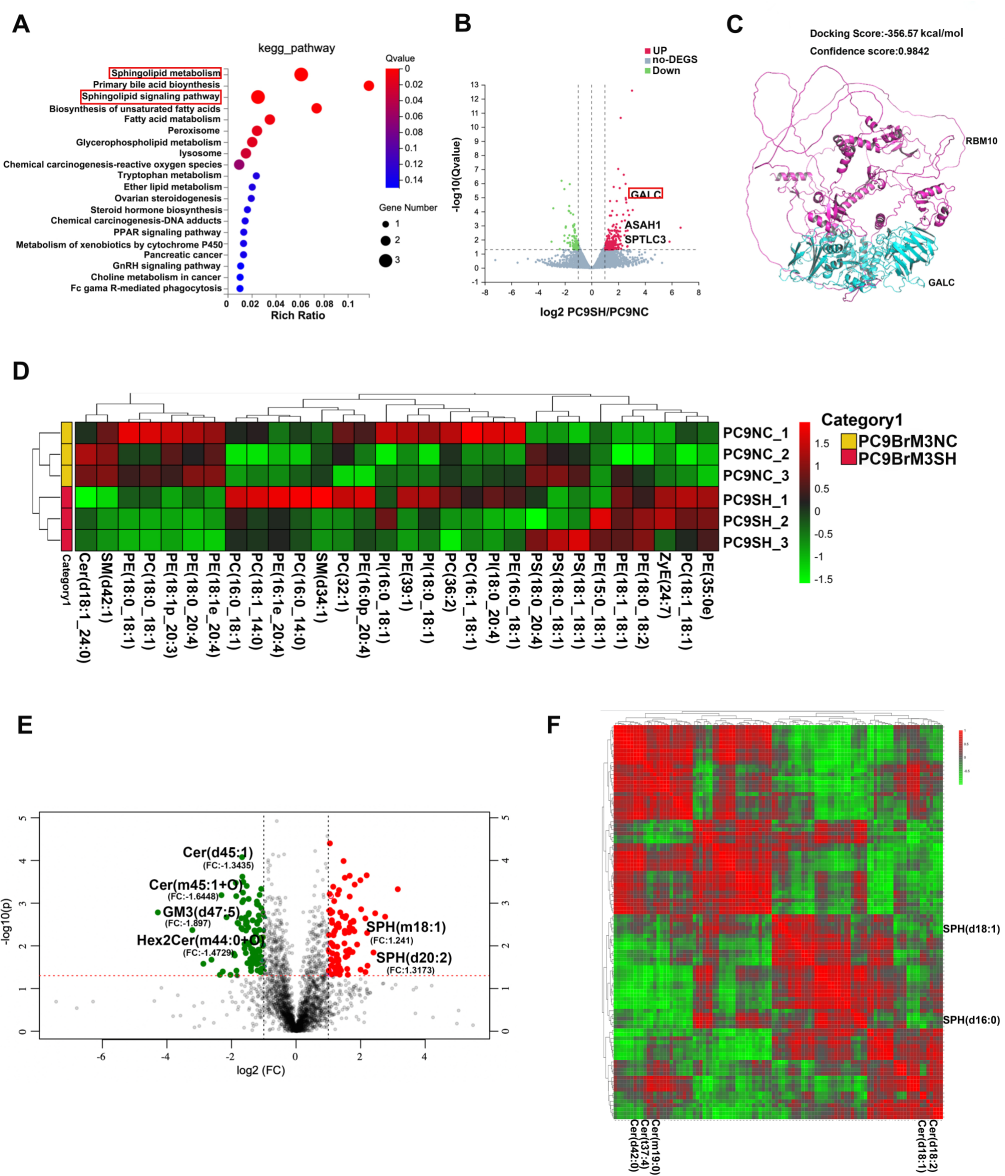
RBM10 regulates the biosynthesis of sphingolipid metabolites. (**A**) KEGG pathway analysis of differentially expressed genes. (**B**) Volcano plot for differentially expressed genes. (**C**) Molecular docking simulation of RBM10 with GALC. (**D**) Metabolite heat map clustering results. (**E**) Volcano plot showing fold changes of metabolites. (**F**) Heat map analysis revealing the correlation between SPH and Cer

### RBM10 regulates the expression of key enzymes in the sphingolipid metabolic pathway and the production of sphingolipid metabolites SPH and ceramide (Cer)

The RNA-seq experiment was conducted collaboratively by BGI-Tech Co., Ltd. (Shenzhen, China). Comparative RNA-seq of PC9sh-NC and PC9shRBM10 cells revealed that RBM10 is associated with sphingolipid metabolism and signaling pathways (Fig. 4A). Differential gene expression analysis identified eight genes associated with lipid metabolism (Fig. 4B and S7A), including *GALC*, *ASAH1*, *PLD1*, *CYP1B1*, *GPDL1*, *SCP2*, *HSD17B4* and *SPTLC3*. *GALC*, *ASAH1* and *SPTLC3* were associated with sphingolipid metabolism. *GALC* exhibited the most significant fold change (*p* < 0.05,|log2| > 1). Protein-protein docking calculations were performed using the HDOCK online tool, which revealed a docking score of − 356.57 kcal/mol and a confidence score of 0.9842, indicating a high likelihood of interaction between GALC and RBM10 proteins (Fig. 4C).

**Fig. 5:**
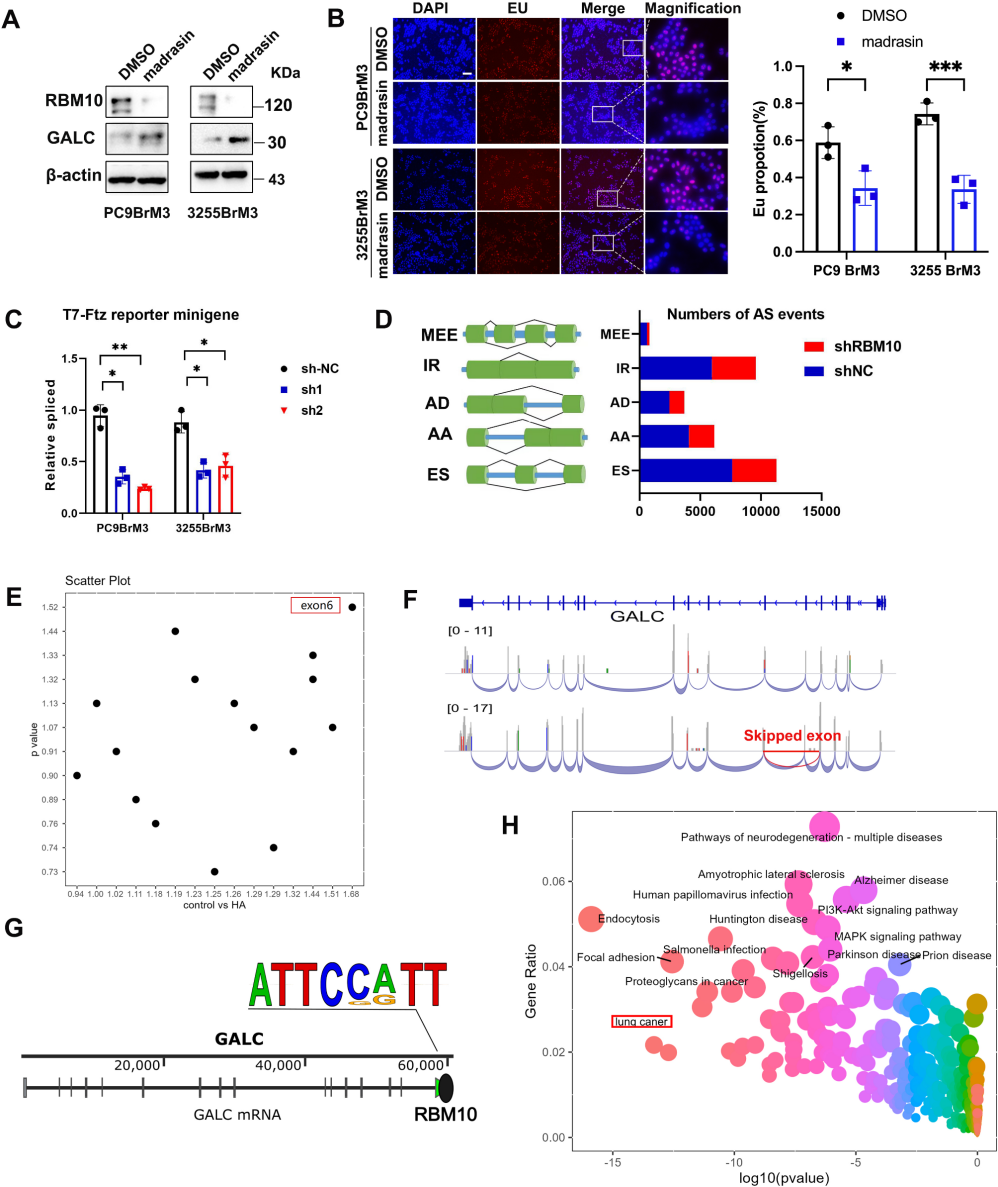
RBM10 regulates GALC, a key enzyme in the sphingolipid metabolic pathway, through alternative splicing. (**A**) WB was used to detect RBM10 and GALC expression after treatment with madrasin. (**B**) New transcripts were detected by EU incorporation assay. (**C**) T7-Ftz reporter minigene assay. (**D**) Nanopore sequencing analysis showed the numbers of altered splicing events in each AS category. (**E-F**) Exon 6 skipping in GALC was identified through a comprehensive analysis. (**G**) The RBM10 binding motif was obtained from MACS2. (**H**) Bubble map annotated by KEGG pathway analysis. *P < 0.05, **P < 0.01

**Fig. 6:**
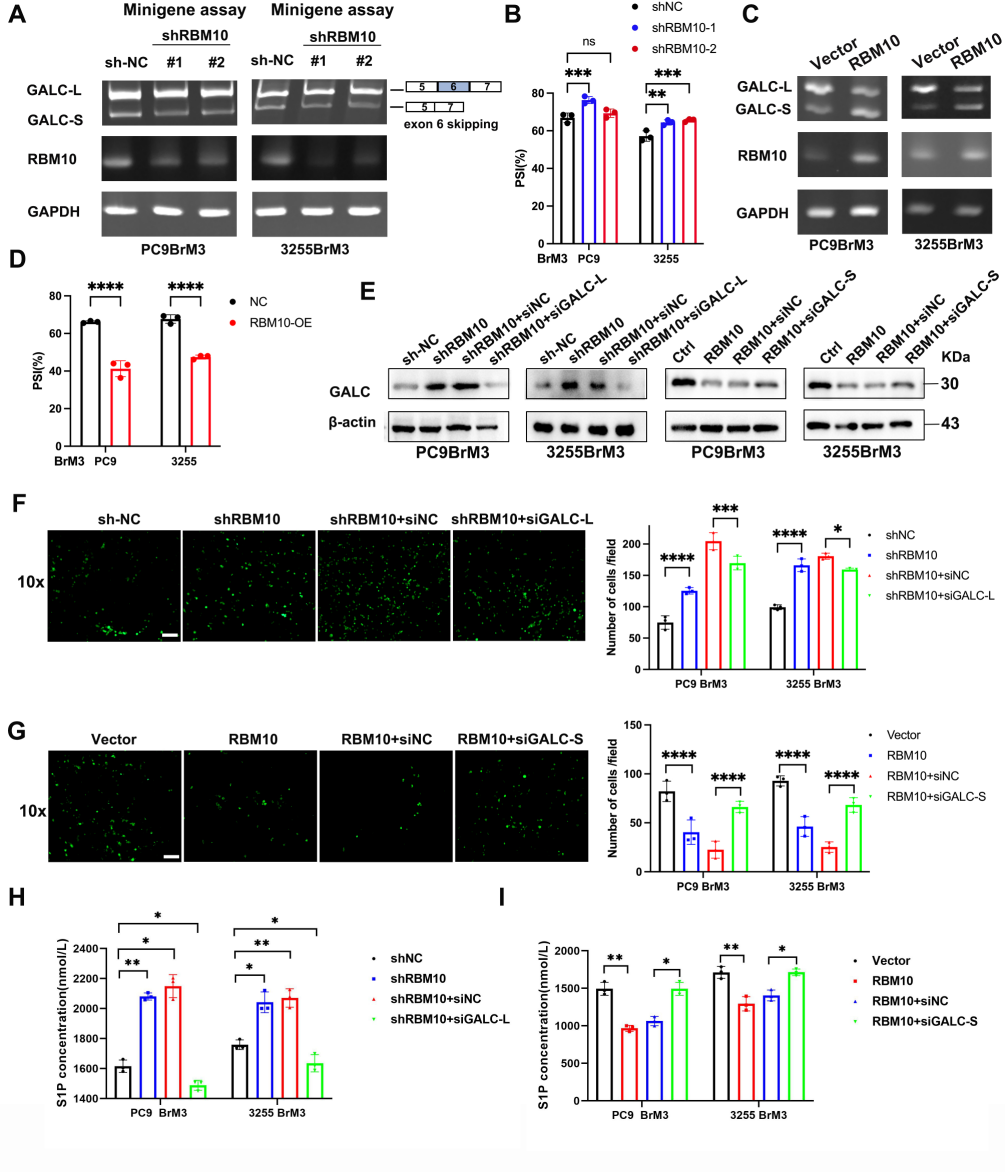
RBM10 inhibits GALC-L and promotes GALC-S to exert tumor-suppressor function. (**A**,** C**) Minigene assays showing the splicing status. (**B**,** D**) Quantification of exon 6 inclusion levels is shown. PSI: percent-spliced-in. (**E**) Expression of GALC proteins after transfection with siGALC-L and siGALC-S were examined by WB. (**F-G**) Trans-BBB migration assay was used to test the transfer capacity under siGALC-L and siGALC-S conditions. (**H-I**) The S1P concentration in tumor cells was quantified using the enzyme-linked immunosorbent assay (ELISA). Scale bar: 10x:50 μm. *P < 0.05, **P < 0.01, **** *P* < 0.0001

To verify the hypothesis that RBM10 influences BBB permeability by regulating sphingolipid metabolism, high-resolution, broad-spectrum, non-targeted lipidomic absolute quantification was performed on RBM10-depleted PC9BrM3 cells. The analysis revealed downregulation of various sphingolipid metabolites, including ganglioside (GM3) (d47:5), Cer (d45:1), Cer (d33:2), Cer (d18:1_24:0), Hex2Cer (m42:3), and Hex1Cer (m44:0 + O), and upregulation of the downstream metabolites of GALC-SPH(m18:1) and SPH(d20:2) (Fig. 4D-E). Lipidomic results also indicated a negative correlation between SPH and Cer (Fig. 4F), which are key metabolites in the sphingolipid metabolic pathway [10, 30]. Cer is a pro-apoptotic signal [31, 32], and SPH is phosphorylated by SK1/2 to generate S1P [33]. S1P is a bioactive lipid mediator, and S1P signaling plays a key role in determining BBB permeability; thus, S1P is a potential therapeutic target in diseases characterized by BBB dysfunction [34]. Cer and S1P constitute sphingolipid rheostats through various sphingolipid metabolic enzymes, which play a key regulatory role in cell signal transduction within the tumor microenvironment [10]. Therefore, RBM10 downregulation may promote brain metastasis by regulating the S1P signaling pathway.

To demonstrate the role of GALC in brain metastases of lung cancer, we conducted a series of experiments. First, data from the Kaplan–Meier plotter (KMplotter) database revealed poorer prognosis in patients with LUAD exhibiting high *GALC* expression (Fig. S6A-C). *GALC* knockdown decreased the number of cells crossing the BBB (Fig. S6D-E) and increased TEER (Fig. S6F). ELISA results also indicated a positive correlation between GALC and S1P (Fig. S6G). In the in vitro model, increased S1P concentration correlated with increased BBB permeability, particularly at 2 µmol/L, where the fluorescence intensity of claudin-5 was the lowest, and the gap between microvascular endothelial cells was the largest (Fig. S6H-I). Based on these findings, GALC was selected as the target of RBM10 for further exploration.

Given that GALC plays an important role in brain metastasis of lung cancer, we next investigated whether RBM10 has a pro-brain metastasis function through its influence on the sphingolipid metabolic pathway. Western blot confirmed that RBM10 regulates GALC (Fig. S7B). ELISA of PC9BrM3 and 3255BrM3 shRBM10 cells revealed increased intracellular GALC expression (Fig. S7C). Moerover, RBM10 knockdown increased the expression of GALC, SPHK1 (SK1), SPHK2 (SK2), and S1PR1-4 (Fig. S7D). Changes in local S1P concentration altered the characteristics of cell membrane receptors and further regulated downstream signaling events. Many effects of S1P in the tumor microenvironment are mediated by its binding to its five G protein-coupled receptors, primarily S1PR1-3, thereby inducing migration and metastasis in various cell types [35]. Psychosine can produce SPH under the catalysis of GALC [9], and SPH can subsequently generate S1P via SK1/2. RBM10 knockdown led to elevated extracellular S1P concentration (Fig. S7E). Moreover, RBM10 knockdown significantly increased S1P concentration in the upper compartment of the BBB model (Fig. S7F). Conversely, RBM10 overexpression decreased S1P concentration (Fig. S7G). Therefore, RBM10 may regulate the production of S1P through the GALC-SPH-SK1/2 signaling axis.

### Revealing the underlying mechanism of brain metastasis induced by RBM10 knockdown in PC9BrM3 and 3255BrM3 cells

RBM10 has been reported to function as a splicing regulator. Does it also regulate GALC through splicing, thereby contributing to the promotion of brain metastases in lung cancer?

To further clarify the impact of RBM10 deficiency on GALC alternative splicing, we treated PC9BrM3 and 3255BrM3 cells with the splicing inhibitor madrasin. Western blot analysis revealed that GALC expression was upregulated (Fig. 5A), this effect is similar to that observed with RBM10 deficiency. In addition, nascent RNA was markedly decreased after madrasin treatment, as shown by the EU incorporation assay (Fig. 5B). To further confirm the general role of RBM10 in splicing, we performed an in vitro splicing assay using the Ftz reporter system with nuclear extracts from cells deficient in RBM10. As shown in Fig. 5C, transcripts were spliced even with a deficiency of RBM10. However, the splicing function was significantly reduced. These findings suggest that RBM10 plays a critical role in splicing and that the upregulation of GALC following RBM10 deficiency is primarily due to severe splicing defects in GALC. These data collectively support that splicing defects in GALC, caused by RBM10 deficiency, are the primary mechanism driving the increased expression of GALC at the RNA level. Nanopore Sequencing analysis suggested that exon skipping was the most frequent splicing event (Fig. 5D). Exon 6 skipping in RBM10-mediated alternative splicing of GALC was most significant (Fig. 5E-F). CLIP-Seq analysis showed the RBM10 binding motif (Fig. 5G). Furthermore, KEGG enrichment analysis of both CLIP-Seq and Nanopore sequencing data was performed, and lung cancer pathways were significantly enriched (Fig. 5H).

To further investigate the functions of different GALC splicing variants, we conducted minigene experiments. The primer sequences were obtained from sequencing data (Table S5). Polyacrylamide gel electrophoresis revealed decreased exon 6 skipping upon RBM10 deficiency (Fig. 6A-D). In addition, selective silencing of GALC splicing variants containing or lacking exon 6 (GALC-L and GALC-S, respectively) was performed in PC9BrM3 and 3255BrM3 cells using siRNA.We found that the RBM10 deficiency increased the expression of GALC-L and subsequently increased the overall expression of GALC (Fig. 6E). Knockdown of GALC-L significantly suppressed the number of cells crossing the BBB compared with the RBM10 deficiency group, whereas silencing of GALC-S had a promoting effect compared to cells transfected with RBM10 (Fig. 6F-G). The above results were consistent with the changes in S1P levels observed between different groups (Fig. 6H-I). These results suggest that GALC-L, but not GALC-S, plays a critical role in brain metastasis.

**Fig. 7:**
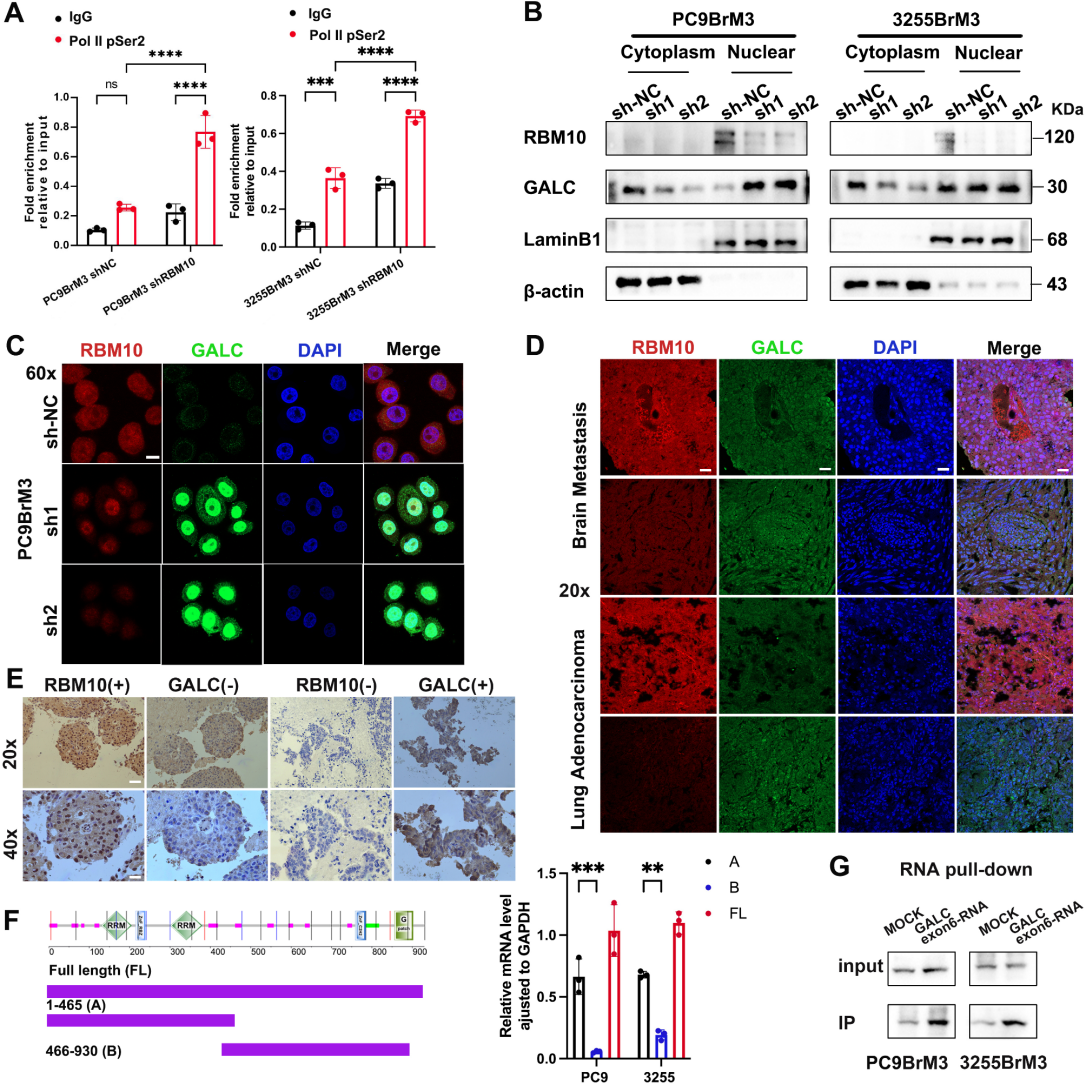
RBM10 deficiency promotes GALC migration to the nucleus. (**A**) ChIP analysis of Pol II-pSer2 binding to the GALC promoter during RBM10 depletion (Pol II-pSer2: RNA polymerase II CTD repeat YSPTSPS antibody). (**B**) WB assay of nuclear and cytoplasmic cellular fractions used to detect the nuclear translocation of GALC. β-actin was used as the cytoplasmic control, and Lamin B1 as the nuclear control. (**C**) Expression of GALC (green) in PC9BrM3 and 3255BrM3 cells when RBM10 was down-regulated. Nuclei counterstained with DAPI (blue). (**D-E**) In vivo co-localization of the RBM10 and GALC. (**F**) RIP assay to verify the binding between RBM10 and GALC. (**G**) RNA pull-down assay showing that RBM10 binds to GALC exon 6 in vitro. Scale bar: 20x:40 μm, 40x:20 μm, 60x:10 μm. **P < 0.01, ***/****P < 0.001

### RBM10 deficiency promotes nuclear translocation of GALC

Phosphorylation of RNA polymerase II CTD repeat YSPTSPS antibody (RNA Pol II Ser2) at the GALC promoter validated that RBM10 exerts splicing regulation through RNA polymerase II (Fig. 7A). RBM10 functions as a splicosome in the nucleus. Through cytoplasmic and nucleus isolation experiments, we found that RBM10 deficiency promotes GALC to enter the nucleus (Fig. 7B). Finally, IF was performed after RBM10 knockdown, which also confirmed this finding (Fig. 7C). The co-localization of RBM10 and GALC was found on IHC and IF (Fig. 7D-E), which further validated the in vitro test. This would provide important evidence for their interaction in vivo. CLIP-seq analysis showed that the RNA binding regions of RBM10 interacting with GALC are mainly within amino acid residues 130 to 205 and 301 to 380, which are RNA recognition motifs and function in binding single-stranded RNAs. We truncated RBM10 in the middle. Using RNA RIP, we found that full-length RBM10, as well as the 1-465 aa and 466–930 aa fragments, associate with GALC (Fig. 7F). This result is highly consistent with the CLIP-Seq assays and supports that the 1-465 aa fragment of RBM10 interacts with GALC to promote GALC splicing. Subsequent RNA pulldown assays demonstrated an interaction between RBM10 and GALC exon 6 RNA (Fig. 7G), further confirming that RBM10 regulates the alternative splicing of GALC exon 6.

### RBM10 plays a crucial role in EGFR-TKI resistance

**Fig. 8:**
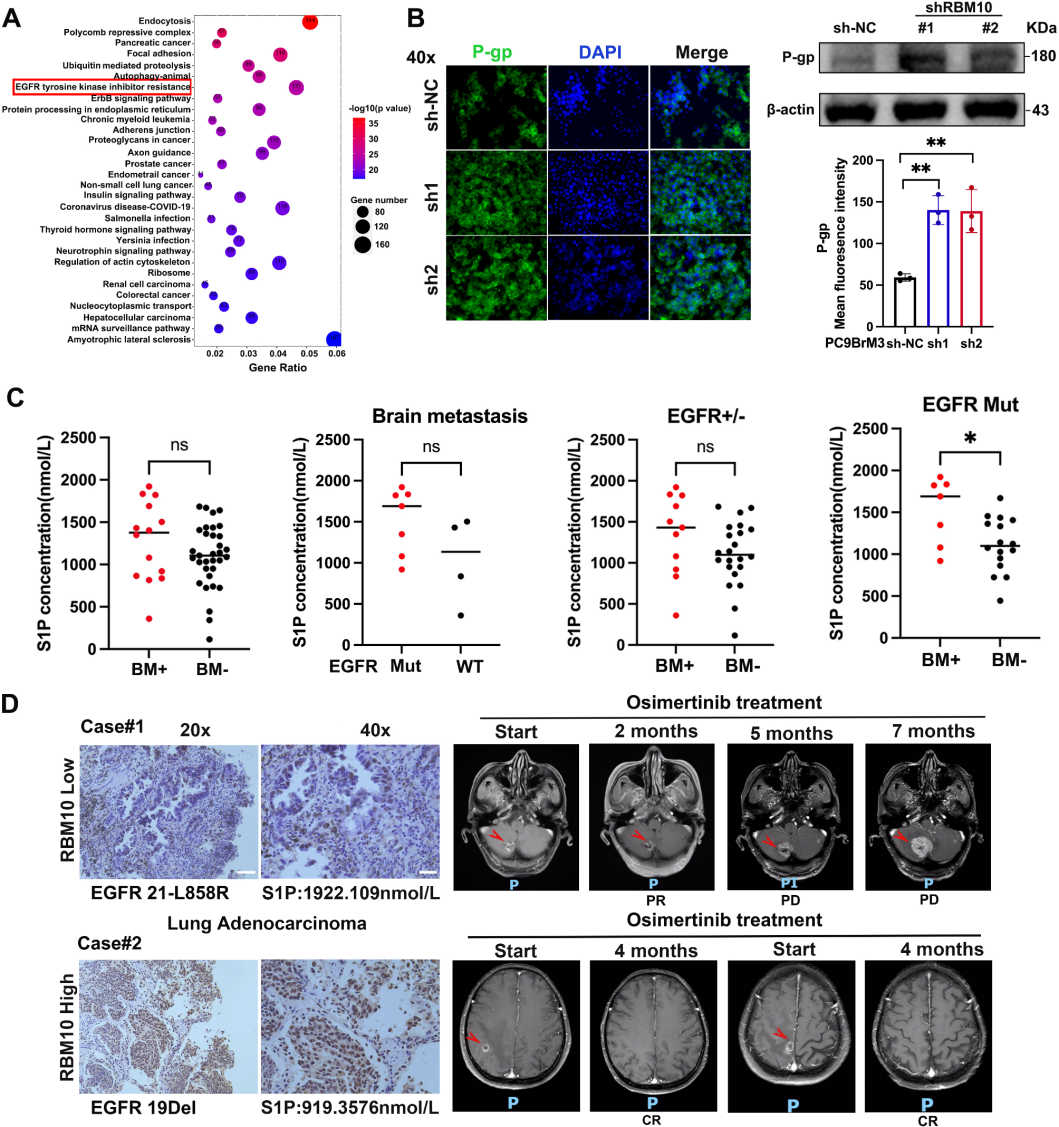
RBM10 deficiency and TKI resistance. (**A**) Dot plot of KEGG annotation results. (**B**) WB and IF were used to detect P-gp. (**C**) The concentration of serum S1P was measured in 47 patients diagnosed with advanced lung adenocarcinoma (data represent medians). (**D**) Two patients with brain metastases harboring EGFR mutations were administered EGFR TKI treatment. Magnetic resonance imaging (MRI) shows brain metastatic lesions (arrowheads). *P < 0.05, **P < 0.01. Scale bar: 20x:40 μm, 40x:20 μm

Pathway analyses of CLIP-Seq, RNA-seq, and Nanopore sequencing data also showed that RBM10 participates in EGFR-TKI resistance (Fig. 8A). Knockdown of RBM10 promoted the expression of P-gp (Fig. 8B), which is one of the resistance markers [36]. In addition, the SK1/S1P/S1PR2 signaling pathway leads to imatinib resistance in chronic myeloid leukemia (CML) [37], and the SK2/S1P/S1PR1/S1PR3 signaling pathway renders the A549 lung cancer cell line resistant to etoposide [38]. This suggests that RBM10 may play an important role in EGFR-TKI resistance via the S1P signaling pathway. In the shRBM10 group, the IC50 values for fingolimod (an S1P inhibitor) and osimertinib (an EGFR-TKI) were higher than those in the control group (Fig. S8A-D). In the in vitro model, TEER was reduced in the combination group (Fig. S8E). Moreover, fingolimod combined with osimertinib significantly reduced brain metastatic capacity (Fig. S8F). Animal experiments demonstrated that the combination therapy significantly reduced brain metastasis, with a marked decrease in lesion fluorescence intensity compared with osimertinib alone (Fig. S8G-H).

Similarly, in the shRBM10 group, the IC50 values for RU-SKI-43 (a potential drug for RBM10 mutation) were lower than those in the control group (Fig. S9A-B). In the in vitro model, TEER was reduced in the combination group (Fig. S9C). Moreover, RU-SKI-43 combined with osimertinib significantly reduced brain metastatic capacity (Fig. S9D). Animal experiments demonstrated that the combination therapy significantly reduced brain metastasis, with a marked decrease in lesion fluorescence intensity compared with osimertinib alone (Fig. S9E). In the Osi + RU-SKI43 combination treatment group, the Ki-67 index was lower (55.38%) compared to the osimertinib-only group (67%) (Fig. S10A). Additionally, the Bax index was higher in the Osi + RU-SKI43 treatment group (53.54%), whereas it was significantly lower (15.6%) in the osimertinib-only group (Fig. S10B). These results suggest that the expression levels of Ki-67 and Bax can serve as indicators for evaluating treatment efficacy in drug-resistant mouse models. The combination treatment group showed more pronounced effects in inhibiting tumor proliferation and promoting cell apoptosis, providing theoretical support for the clinical use of a combination therapy involving RU-SKI43 and osimertinib.

Subsequently, we collected a total of 47 clinical samples. The pathological type was identified as LUAD, stage IIIB/IV, and the molecular detection results were confirmed. The mutation status and brain metastasis of the patients are presented in Table S6. In patients with EGFR mutations, the concentration of S1P in the brain metastasis group was found to be elevated. In 6 of 7 patients with brain metastasis, the serum S1P concentration exceeded 1 µmol/L (Fig. 8C). Moreover, Case 1 involved a patient harboring an *EGFR 21-L858R* mutation and exhibiting low RBM10 expression before osimertinib treatment. This patient had a partial response (PR) for 3 months, followed by early progression. In Case 2, the patient carried an EGFR 19Del mutation and exhibited high RBM10 expression, achieving complete remission (CR) after 4 months of osimertinib treatment (Fig. 8D). The remaining 5 brain metastasis patients showed partial response (PR) during the initial evaluation of osimertinib treatment but experienced brain metastasis recurrence or developed new metastatic lesions within 8 to 13 months. The above results demonstrate that RBM10 deficiency reduces the efficacy of TKI treatment and positions tumor cells within a favorable niche in the brain microenvironment.

**Fig. 9:**
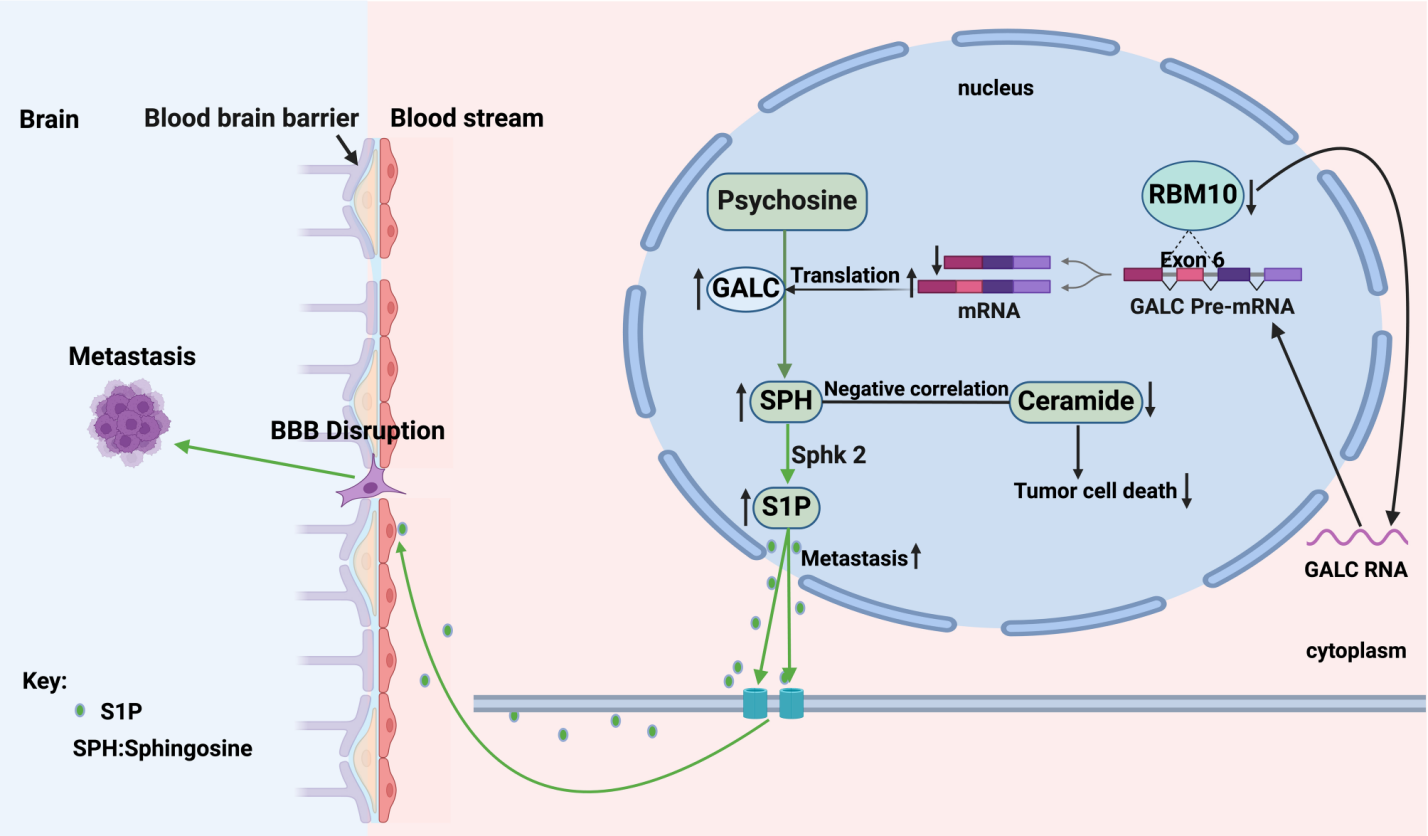
A schematic diagram of RBM10 deficiency promoting the development of brain metastases through regulation of sphingolipid metabolism

Collectively, these findings demonstrate that RBM10 deficiency impacts the GALC-SPH-SK2-S1P signaling axis in the sphingolipid metabolism pathway, leading to elevated S1P concentration and increased BBB permeability (Fig. 9).

## Discussion

RBM10 expression is downregulated in lung adenocarcinoma (LUAD) and is significantly associated with tumor cell proliferation and apoptosis [18, 19]. Our previous research demonstrated a link between RBM10 deficiency and lung cancer metastasis [20]. Foggetti et al. reported that the *RBM10* mutation promoted tumor growth in mice with *EGFR* mutations and Trp53 deficiency [39]. Nanjo et al. discovered that such co-mutations affect the selective splicing of Bcl-x, an apoptosis regulator, and are more likely to lead to the development of resistance to EGFR-TKI [40]. Zhang et al. found that RBM10 can regulate alternative splicing of EIF4H, influencing proliferation and metastasis [41]. These results explore the relationship between co-mutation of *RBM10* and *EGFR* and tumor progression, drug resistance, and their underlying mechanisms. However, the specific role of co-mutation of *RBM10* and *EGFR* in brain metastasis among patients remains unexplored.

WES on five LUAD primary tumor–normal pairs from patients who later developed brain metastasis showed that truncating mutations in RBM10 occur in patients with lung cancer brain metastases. RBM10 truncating mutations lead to low expression of RBM10. NMD is probably the best-characterised eukaryotic RNA degradation pathway. Through intricate steps, NMD factors recognise and degrade mRNAs with translation termination codons positioned in abnormal contexts [42]. UPF1 is considered the principal NMD factor because it is central to most (if not all) steps, from recognition of the PTC-containing mRNAs to their degradation. RBM10 truncating mutations lead to low expression and functionality via the NMD pathway and affect prognosis.

To investigate the mechanisms underlying brain metastasis in lung cancer, we established both in vivo and in vitro blood-brain barrier (BBB) models. First, we developed six in vitro BBB models using three cell types with different co-culture contact methods. Among these, we selected the model with low permeability and high tightness for subsequent experiments, making it highly suitable for studying brain metastases. Additionally, we screened cell lines with a propensity for brain metastasis from animal models. Both in vivo and in vitro models demonstrated that RBM10 knockdown enhances transmigration of tumor cells across the BBB and increases brain metastasis frequency in nude mice.

Our previous RNA-seq analysis revealed the potential regulatory role of RBM10 in modulating GALC expression within the sphingolipid metabolism pathway. GALC expression in circulating tumor cells is significantly associated with distant metastasis, tumor burden, and treatment-related adverse events in non-small cell lung cancer [43]. In lung cancer brain metastasis model, GALC can also promote the cancer cells to penetrate the BBB. The lipidomic analysis further showed that RBM10 knockdown decreased Cer levels and increased SPH. It has been proposed that modulation of GALC expression can regulate Cer levels, with its upregulation decreasing Cer levels and promoting tumor cell proliferation [44]. Therefore, we hypothesised that RBM10 might influence sphingolipid metabolism by interacting with GALC. SPH is the precursor for S1P production. The balance between Cer and S1P, known as the sphingolipid rheostat, is crucial in cell signaling [45]. Mover, higher plasma levels of S1P activate S1P2, causing cytoskeletal rearrangement and consequently BBB disruption [12]. Thus, a balance of S1P receptor signaling is important for the modulation of BBB integrity. Our results suggested that RBM10 affects the levels of S1P by regulating GALC by influencing a series of key enzymes downstream in sphingolipid regulation. S1P exerts a potent stimulatory effect on sustained cell proliferation, whereas Cer can induce programmed cell death (apoptosis) [45, 46]. Our in vitro experiments confirmed that S1P enhances BBB permeability, consistent with previous reports [11, 12]. Moreover, Increased expression of S1P receptors and elevated S1P concentrations in tumor cells enhance their metastatic capacity [35]. With the addition of S1P inhibitors to the* in vitro* BBB model, the number of tumors crossing the BBB decreased, and TEER values increased, indicating a reduction in BBB permeability (Fig. S8E-F). The results of the above experiments indicate that S1P plays a crucial role in maintaining BBB permeability.

Our study is the first to elucidate the mechanism of RBM10 in brain metastasis of *EGFR 19Del* and *L858R* LUAD. Combined Nanopore sequencing (third-generation sequencing) and CLIP-Seq analysis revealed that RBM10 deficiency inhibits exon 6 skipping during GALC splicing, leading to upregulated GALC-L expression (GALC) and increased S1P synthesis. Through alternative splicing of GALC, RBM10, as a splicing factor, downregulates GALC expression, leading to decreased S1P concentration and subsequent inhibition of brain metastasis formation in lung cancer.

RNA polymerase II plays a critical role in regulating alternative splicing [47, 48]. Based on the results of this experiment, RBM10 directly impacts the alternative splicing of GALC by interacting with RNA polymerase II. RBM10 is located in the nucleus, while GALC is located in the cytoplasm. How does RBM10 regulate GALC? We found that GALC enters the nucleus to function when RBM10 expression is low. RBM10 controls GALC nucleocytoplasmic shuttling. RIP showed that the RNA binding regions between RBM10 and GALC are mainly in the ranges of 301 to 380 aa and 130 to 205 aa, which are RNA recognition motifs and have the function of binding single-stranded RNAs as predicted by CLIP-Seq. Then, RNA pulldown further confirmed that RBM10 bound to GALC exon 6 RNA. Overall, these findings highlight a pivotal role for RBM10 in brain metastasis, demonstrating that RBM10 deficiency upregulates the GALC-SPH-SK2-S1P signaling pathway, thereby promoting brain metastasis.

We further validated the inhibitory effect of fingolimod, an S1P inhibitor, and RUSKI-43, a potential drug for RBM10 mutation, on brain metastasis and demonstrated the synergistic efficacy when combined with osimertinib in suppressing brain metastasis. The findings of this study suggest that targeting S1P, which is regulated by RBM10, could be a promising therapeutic strategy for patients with lung cancer associated with RBM10 deficiency. In patients with EGFR mutations, S1P concentration was significantly higher in the peripheral blood of those with brain metastasis. Patients with low RBM10 expression and high S1P levels had a poor response to osimertinib. The results of the above studies suggest that RBM10 deficiency may impair the response to EGFR inhibitor treatment in EGFR-mutant LUAD. These findings are consistent with previously reported literature [40]. However, additional clinical samples are needed for further validation.

One of the limitations of this study is the potential variability in intracardiac injection, which may have influenced the efficiency of brain metastasis formation. Although we aimed for precise left ventricular injections by observing the blood reflux velocity before injection, occasional misplacements into the right ventricle could have contributed to higher lung signals and reduced brain metastases. This technical variability highlights the need for improved methodologies in future studies. To enhance the accuracy of intracardiac injections, we plan to incorporate real-time ultrasound guidance or fluorescence-assisted techniques to ensure consistent delivery into the left ventricle. These refinements will help minimize injection-related inconsistencies and improve the reliability of brain metastasis models. Moreover, the PC9BrM3 shRBM10 cell line exhibited a 100% success rate in the brain metastasis model established through intracranial orthotopic injection, making it a reliable tool for developing and screening anticancer agents. However, the low tumor formation rate of PC9BrM3 hinders its suitability for subsequent drug treatment experiments, necessitating further screening for cell lines with a higher propensity for brain metastasis in nude mice.

## Conclusion

In summary, we have successfully established an *EGFR 19Del* and *L858R* LUAD brain metastasis model and elucidated the role and mechanism of RBM10 deficiency in brain metastasis formation. Our findings contribute to a better understanding of the underlying mechanisms and may provide insights for future research on strategies to mitigate brain metastasis in LUAD. Additionally, this study suggests a potential therapeutic target that warrants further investigation.

## Electronic supplementary material

Below is the link to the electronic supplementary material.

Supplementary Material 1

## Data Availability

The datasets supporting the conclusions of this article are available in the NCBI SRA and NCBI GEO repository, unique persistent identifier and hyperlink to datasets in https://dataview.ncbi.nlm.nih.gov/object/PRJNA1180963?reviewer=8qngr8sibfcsfhe4uphcqo7hjq. The CLIP-Seq sequencing raw data has been deposited in NCBI SRA with an accession number PRJNA1180963. The RNA sequencing raw data has been deposited in NCBI GEO with an accession number GSE280915.

## Abbreviations

BBB: blood-brain barrier
BMFS: brain metastasis-free survival
BLI: bioluminescence imaging
Cer: ceramide
CHX: cycloheximide
ChIP: Chromatin immunoprecipitation
CML: chronic myeloid leukemia
CR: complete response
Del: deletion mutations
ELISA: enzyme-linked immunosorbent assay
EGFR: Epidermal growth factor receptor
EU: 5-ethynyluridine
GALC: galactosylceramidase
GFAP: glial fibrillary acidic protein
GLUT-1: glucose transporter protein-1
IHC: immunohistochemistry
IF: immunofluorescence
KMplotter: Kaplan-Meier plotter
LUAD: lung adenocarcinoma
Mut: mutation
NMD: nonsense-mediated mRNA decay
P-gp: p-glycoprotein
PR: partial response
PTC: premature termination codons
RBM10: RNA binding motif protein 10
RIP: RNA immunoprecipitation
RNA Pol II Ser2: RNA polymerase II CTD repeat YSPTSPS antibody
S1P: sphingosine 1-phosphate
SPH: Sphingosine
SK1/2: sphingosine kinase 1/2
TCGA: The Cancer Genome Atlas
TEER: transendothelial electrical resistance
TKI: tyrosine kinase inhibitor
WT: wild-type
WES: whole-exome sequencing
ZO-1: zonula occluden-1
