# Supplementary material for: RBM10 deficiency promotes brain metastasis by modulating sphingolipid metabolism in a BBB model of EGFR mutant lung adenocarcinoma
Source: J Exp Clin Cancer Res. 2025 Mar 11;44:95. doi: 10.1186/s13046-025-03347-1 (PMC11895392; doi:10.1186/s13046-025-03347-1)
Supplement: Supplementary file 1 — Supplementary Material 1 [file 13046_2025_3347_MOESM1_ESM.docx]

**RBM10** **Deficiency Promotes Brain Metastasis** **by Modulating Sphingolipid Metabolism in a BBB Model of *EGFR* Mutation** **Lung Adenocarcinoma**

Gang Xu^1#^, Bo An^1#^, Ruqiong Wang^1^, Bo Pan^1^, Huiting Hao^2^, Xingmei Ren^1^, Zihan Jing^1^, Weitong Gao^1^, Yajie Li^1^, Yan Jin^3^, Enguang Lin^4^, Lihua Shang^1*^, Dexin Jia^1*^, Yan Yu^1*^

**Supplementary methods**

**ELISA assays**

Human S1P ELISA KITs were bought from MEIMIAN Industrial CO., LTD (China). The assays were carried according to the manufacturer’s protocol. Written informed consent was obtained from all participants. The use of serum from the Third Hospital of Harbin Medical University was approved by the Third Clinical Ethics Committee of Harbin Medical University. All patients provided informed consent.

**5-ethynyluridine (EU) Labeling of Cultured Cells**

PC9BrM3 and 3255BrM3 cells were grown on a culture bottle in DMEM supplemented with 10% bovine calf serum, penicillin, and streptomycin. EU (R0309S, Beyotime, China) was added to the complete culture medium from a 100 mM stock in DMSO. After EU labelling, cells were washed with PBS and fixed.  The fixed cells were rinsed once with TBS. Next, the nuclei were stained with DAPI. The cells were then imaged using a fluorescence microscope.

**Cell–Cell Adhesion Assay**

HCMEC and SVG cells were seeded in a 6-well plate and allowed to form a confluent monolayer for the appropriate time. Then, the tops of the monolayers were seeded with cancer cells labelled with 5 μM of CellTracker Green CFMDA. The cells were allowed to incubate for 30 minutes. The media was aspirated and cells were washed with PBS twice to remove the nonadherent cells. The fluorescent tumor cells were imaged and the numbers of cells were counted per field.

**4-Hour Liquid Leakage Assay**
First, six in vitro blood-brain barrier (BBB) models were established. After 72 hours, the culture medium in both the upper and lower chambers was refreshed, followed by a 4-hour incubation in a cell culture incubator. Subsequently, the liquid level difference between the two chambers was measured. A difference greater than 5 mm indicates high BBB integrity.

**Cell proliferation assays**

Cells were counted and seeded in 96-well plates (2×103 cells/well). After incubation for 24 h, 10 μl of Cell Counting Kit-8 (CCK-8, Dojindo, Kumamoto, Japan) was added to the culture medium and incubated for 2.0 h at 37 °C. Then, the optical density (OD) value at 450 nm was measured by using SpectraMax Paradigm (Molecular Device, CA, USA). All of the values were standardized by comparison with the data from the untreated cells. Three independent experiments were performed.

**5-Ethynyl-2′-deoxyuridine (EdU) incorporation assay**

The PC9 and PC9BrM3 cells were inoculated into 24-well plates. EdU kit (RiboBio, Guangzhou, China) was used for labeling cells following the manufacturer’s instructions. Photographs were taken using an inverted fluorescent microscope (Leica Microsystems Inc., USA), and the experiment was repeated three times.

**Wound healing assay**

The PC9 and PC9BrM3 cells were seeded into 6-well plates. When the cell density reached over 80%, a 200μl pipette tip was used to scratch three separate wounds through the cells, moving perpendicular to the line. The cells were then gently rinsed twice with PBS to remove floating cells and cultured in the medium containing 0.5% FBS serum for 48 hours. Images of the scratches were taken using an inverted microscope (Olympus, Tokyo, Japan) at ×10 magnification at 0 and 48 h of incubation. The experiments were run in triplicate.

**Transwell assay**

In brief, 3×105 cells were resuspended in 300ul serum-free medium and then seeded in the upper chamber (BD Biosciences, New Jersey, USA) pre- coated with or without 40 μl diluted Matrigel, while 700 μl medium supplemented with 10% FBS was added in the lower chamber. After 48 h, cells on the top surface of the microporous membrane were wiped off with a cotton swab. The remaining cells were fixed with 4% paraformaldehyde, stained with 0.1% crystal violet, and counted per 3 random fields for each assay under a microscope (Leica Microsystems Inc., USA). The data are obtained from three independent experiments.

**Supplementary figures**


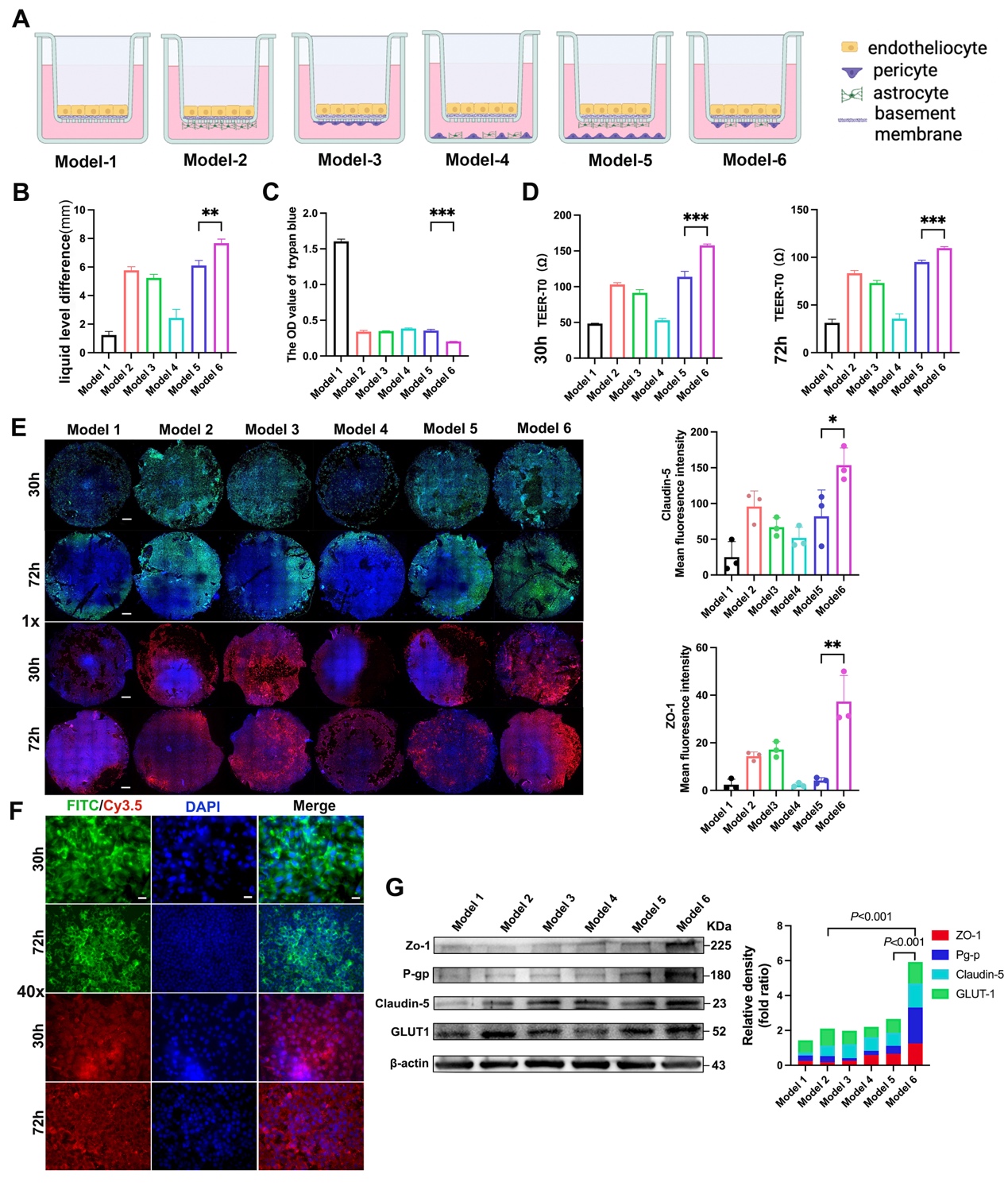


**Figure S1.**

Construction of *in vitro* BBB models.

(A) Schematic illustration of *in vitro* models for studying the BBB. (B) Results of leakage experiment after 4 hours. (C-D) Comparison of permeability and compactness of different models (T0: TEER of the transwell model without cells). (E) Six panoramic scanning immunofluorescence images of the BBB obtained *in vitro* (Claudin-5: green, ZO-1: red). Scale bar: 1x: 600μm. (F) Model 6 was observed at a magnification of 40x. Scale bar: 20μm. (G) Comparison of expression of BBB⁃related proteins in different models. The results were represented as mean ± SD, **P*<0.05, ***P* < 0.01, ****P*<0. 001. Each experiment was repeated three times.


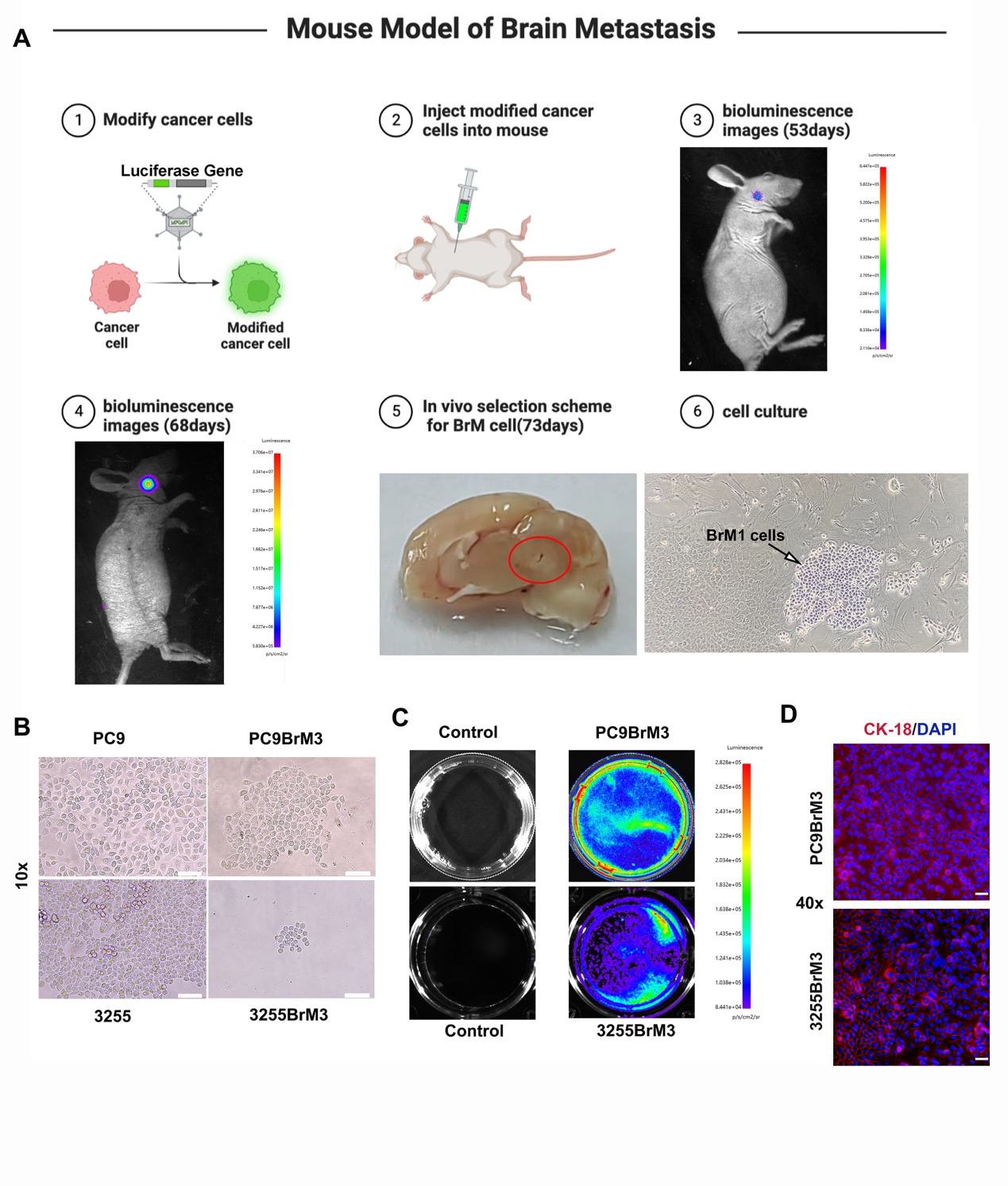


**Figure S2.**

Establishment of BrM3 cell line.

(A) Establishment of a mouse model of brain metastasis. (B-E) Identification of the PC9BrM3 and 3255BrM3 cell lines. Scale bar: 10x:50μm, 40x:20μm.

**
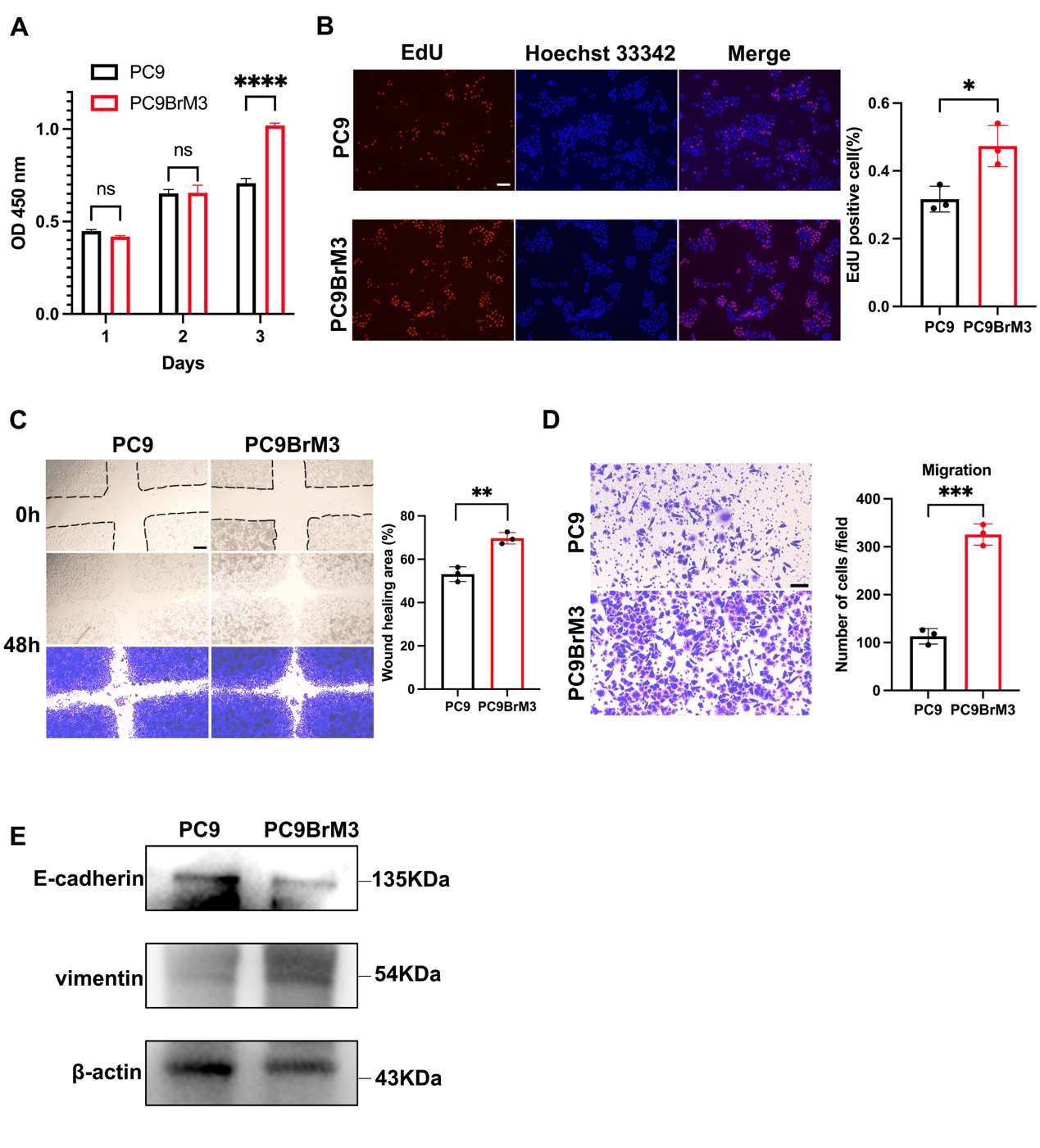
**

**Figure S3** Differences in cellular biology between PC9BrM3 and PC9. (A) Cell proliferation was examined by CCK8 (The results were represented as mean ± SD, **P* <0.05, Scale bar is 100μm.); (B) EdU assays (scale bar is 100μm, cells synthesizing DNA stained with EDU (red), Nuclei counter stained with Hoechst 33342 (blue); (C) A wound healing assay was used to test the migration capacity of PC9BrM3 cells. The cells migrating into the wounded areas were photographed at 0h and 48h. The results were represented as mean ± SD, ***P*<0.01, Scale bar is 100μm. (D) The migration capacity of PC9BrM3 cells was also examined by Transwell assays. The results were represented as mean ± SD, ****P*<0.001, Scale bar is 100μm. (E) Western blot analysis of the EMT markers (E-cadherin, Vimentin) in PC9 and PC9BrM3cells.


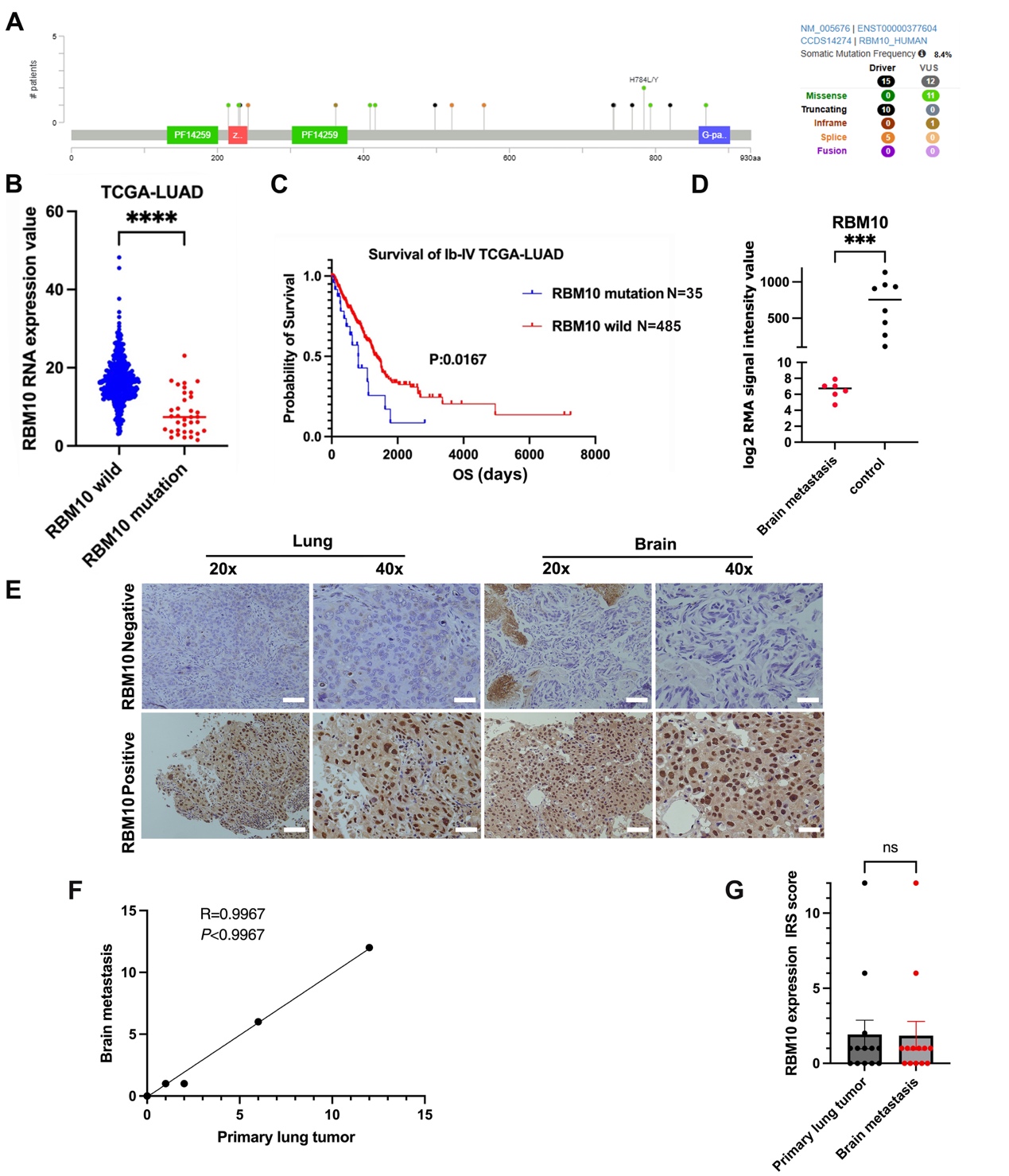


**Figure S4.**

Location distribution, mutation types, expression, and survival analysis of RBM10 in different databases, along with immunohistochemistry analysis. (A) Location distribution and mutation types of RBM10 mutations in the cBioPortal database. (B) Expression of RBM10 in the RBM10 mutant group and RBM10 wild-type group in the TCGA database. (C) Survival analysis of stage IB-IV lung adenocarcinoma patients with RBM10 mutation and wild-type group in the TCGA database. (D) Expression of RBM10 in patients with and without brain metastases in the HCMDB database (data represent medians, n=14, *p*=0.0010). ****P*<0.001. (E) Representative images of RBM10 immunohistochemistry analysis of LUAD and brain metastasis tissue samples. Scale bar: 20x:40μm, 40x:20μm. Relative RBM10 staining intensities from low to high were scored 0, 1, 2, and 3. (F-G) The correlation and expression of RBM10 in the primary lung lesions and brain metastases.


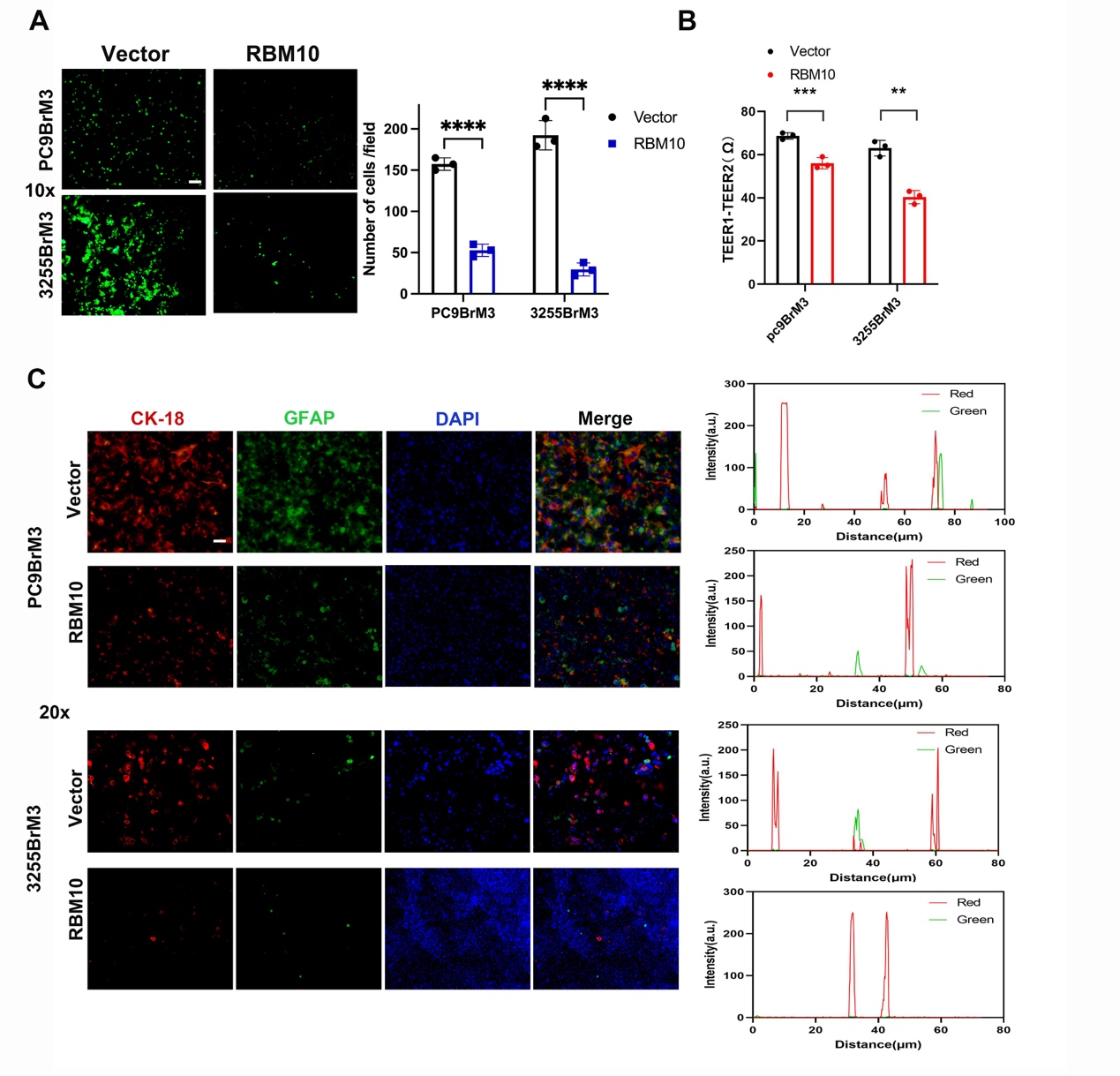


**Figure S5.**

RBM10 inhibits the migration of PC9BrM3 and 3255BrM3 cells in an *in vitro* BBB model. (A) Trans-BBB migration assay was used to test the transfer capacity under RBM10 overexpression conditions. (B) TEER in the upper compartment of the BBB model was measured (TEER1 is the resistance measured after successful BBB construction; TEER2 is the resistance measured after the addition of tumor cells). (C) The number of tumor-associated astrocytes was observed (Red: CK-18, Green: GFAP).

The results were represented as mean ± SD, **P*<0.05, ***P* < 0.01, ****P*<0. 001. Each experiment was repeated three times. Scale bar: 10x:50μm.


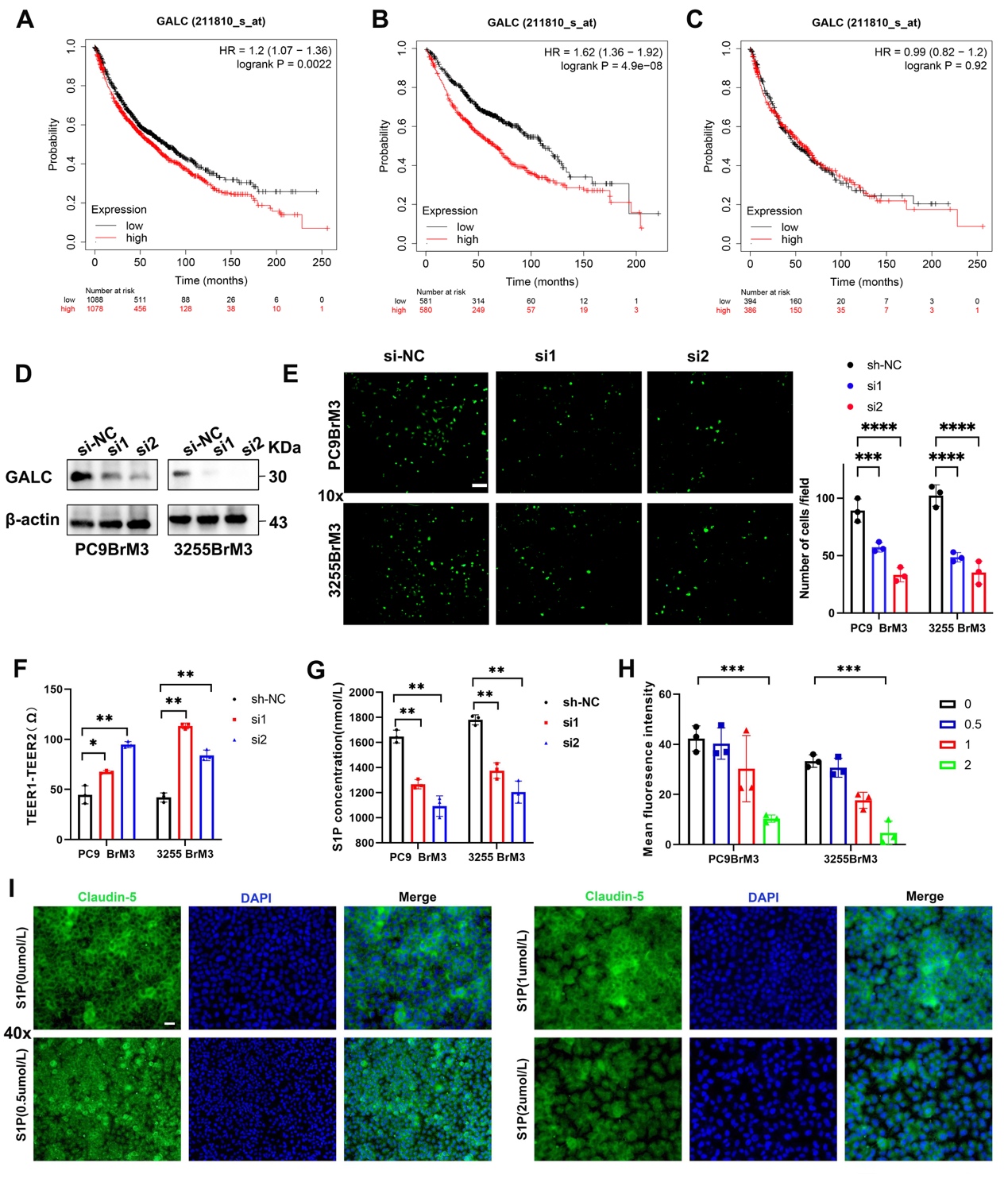


**Figure S6.**

GALC is associated with poor prognosis, regulates S1P production, and elevated S1P levels disrupt the integrity of the BBB.

(A-C) The Kaplan-Meier plotter database was searched for the overall survival of lung cancer patients. A: adenocarcinoma and squamous cell carcinoma, B: adenocarcinoma, C: squamous cell carcinoma. (D) WB was used to detect GALC. (E) Trans-BBB migration assay was used to test the transfer capacity under siGALC conditions. (F) TEER in the upper compartment of the BBB model was measured. (G) Concentrations of S1P in the upper compartment of the BBB model. (H-I) Expression of Claudin-5 proteins with the different S1P concentrations was examined by immunofluorescence. **P*<0.05, ***P*<0.01, ****P*<0.001. Each experiment was repeated three times. Scale bars:
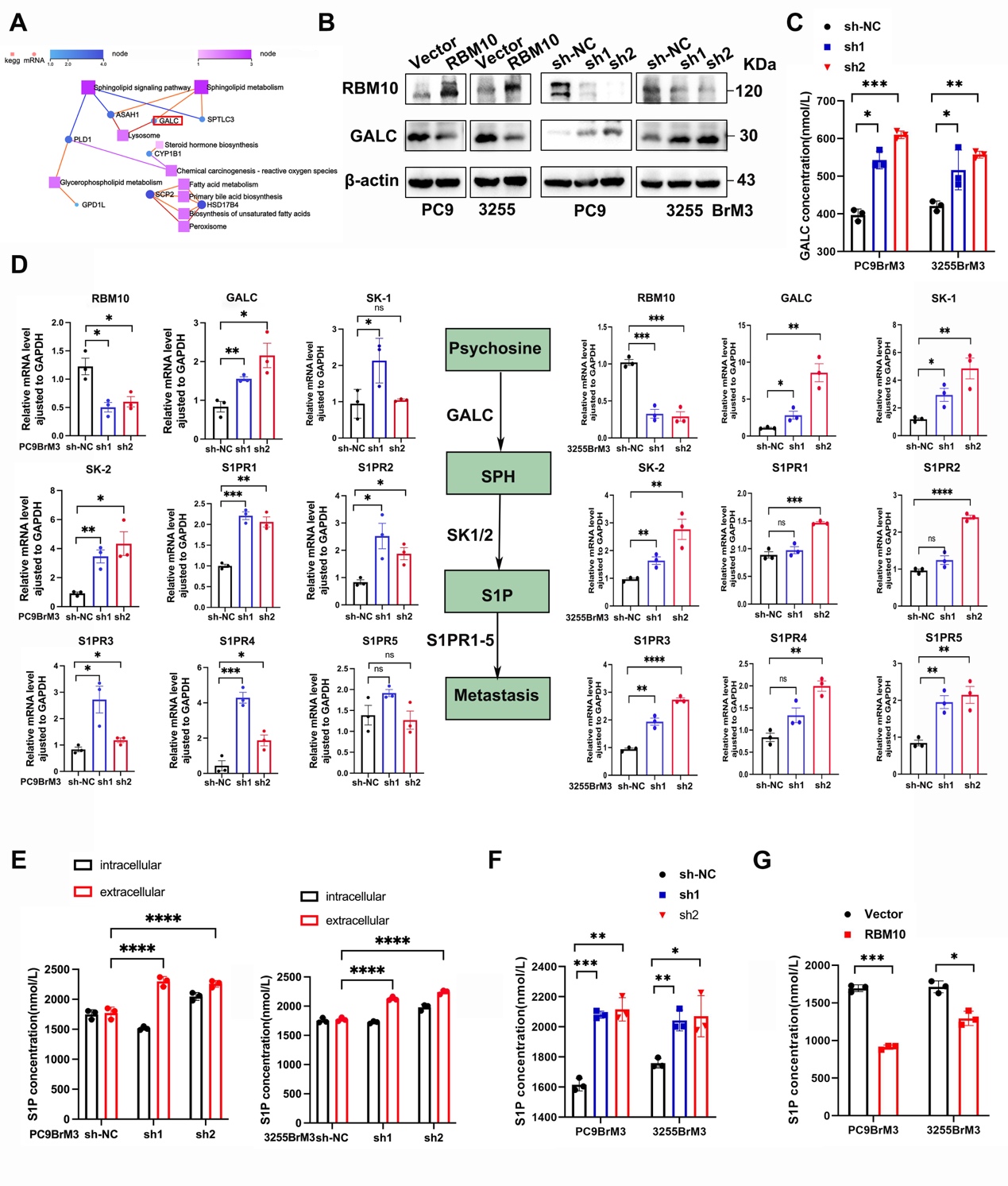


**Figure S7.**

RBM10 regulates sphingolipid metabolism.

(A) KEGG network diagram. (B) WB was used to detect RBM10 and GALC. (C) The GALC concentration in tumor cells was quantified using the enzyme-linked immunosorbent assay (ELISA). (D) Quantitative real-time PCR was performed to assess the mRNA expression of sphingolipid metabolism and S1P receptors in tumor cells. The obtained data were normalised to the expression levels of the housekeeping gene GAPDH (the results are represented as mean ± SD). (E) Concentrations of S1P in tumor cells and the extracellular fluid. (F-G) S1P concentration in the upper compartment of the BBB model. **P*<0.05, ***P*<0.01, ****P*<0.001, *****P*<0.0001. Each experiment was repeated three times.


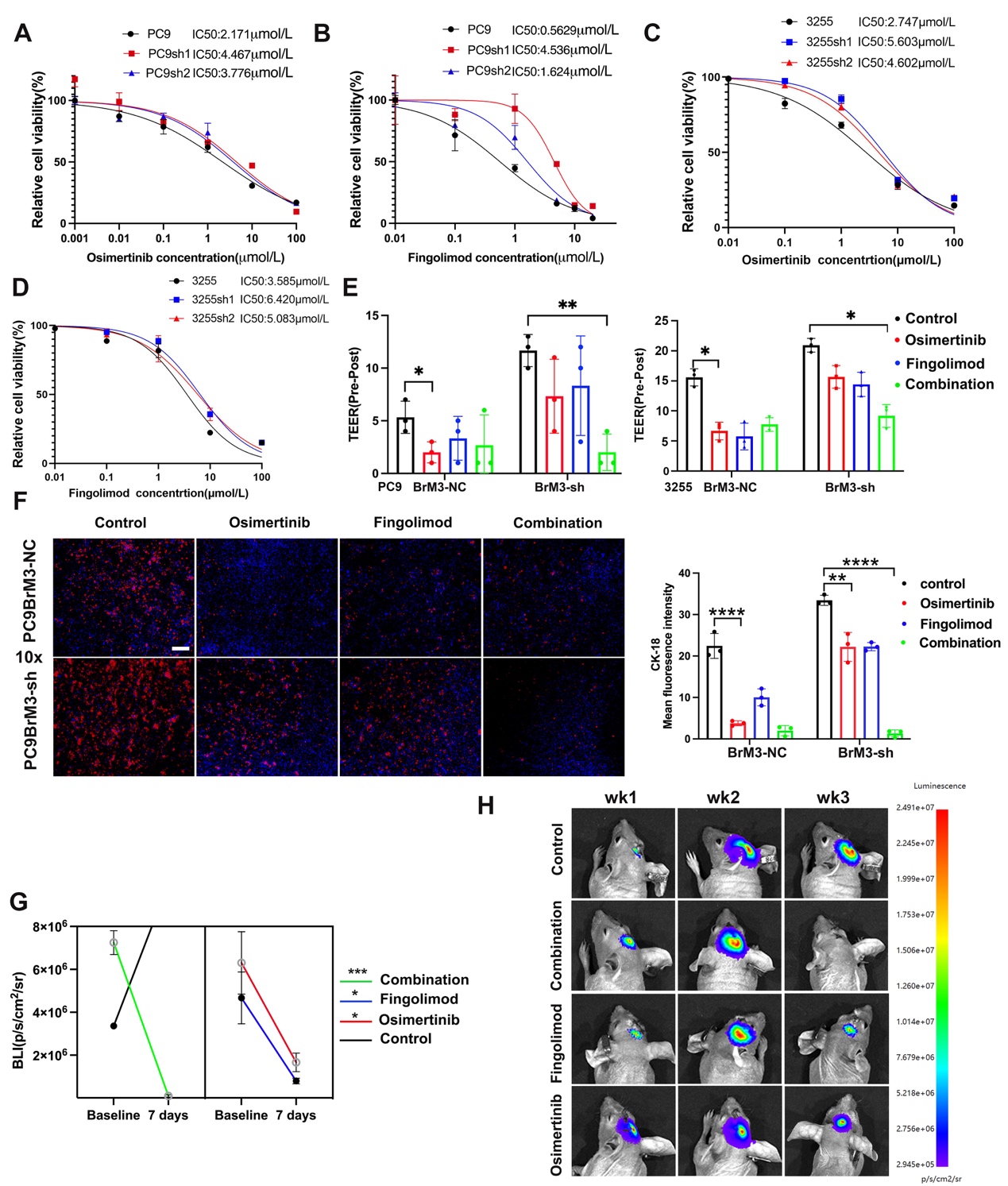


**Figure S8.**

The combination of an S1P inhibitor (fingolimod) and osimertinib exhibits a synergistic effect in both *in vitro* and *in vivo* models.

(A-D) Cell viability was assessed in cells exposed to the indicated concentrations of fingolimod and osimertinib for 24 hours. The experiment was repeated three times. (E) TEER was measured in the upper compartment of the BBB model before and after drug administration (The results are represented as mean ± SD). (F) Tumor cells were added to the *in vitro* BBB model to assess the efficacy of fingolimod combined with osimertinib in inhibiting tumor cell transmigration across the BBB. (G-H) BLI and quantification of the relative fold change of mice receiving an intracranial injection of RBM10-knockdown PC9BrM3 cells (data are mean ± SD. Each group contains three mice). **P*<0.05, ***P*<0.01, ****P*<0.001, *****P*<0.0001. Scale bar: 10x:50μm.


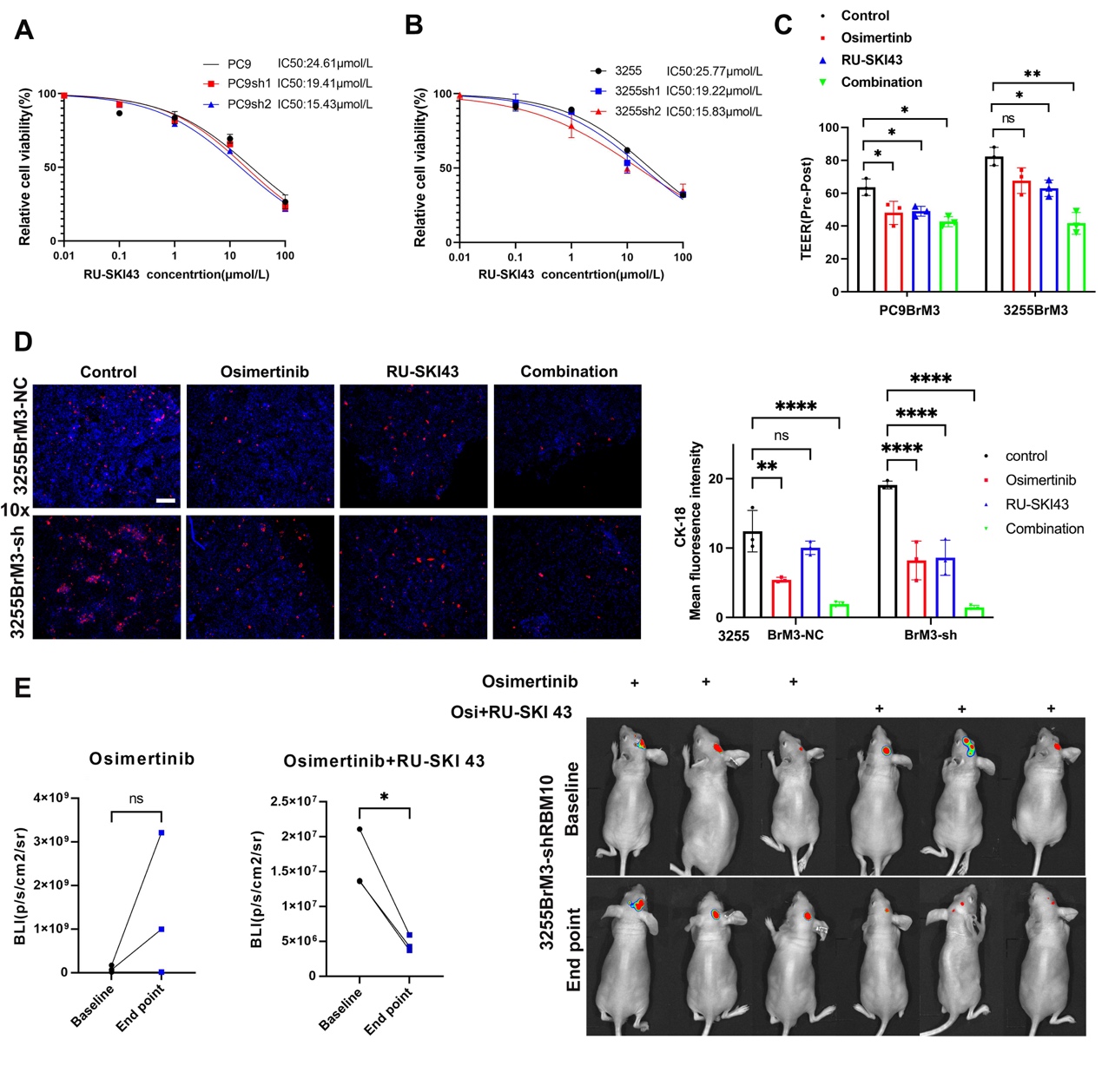


**Figure S9.**

The combination of an RBM10 mutant inhibitor (RU-SKI43) and osimertinib exhibits a synergistic effect in both *in vitro* and *in vivo* models.

(A-B) Cell viability was assessed in cells exposed to the indicated concentrations of RU-SKI43 and osimertinib for 24 hours. The experiment was repeated three times. (C) TEER was measured in the upper compartment of the BBB model before and after drug administration (the results are represented as mean ± SD). (D) Tumor cells were added to the *in vitro* BBB model to assess the efficacy of RU-SKI43 combined with osimertinib in inhibiting tumor cell transmigration across the BBB. (E) BLI and quantification of the relative fold change of mice receiving an intracranial injection of RBM10-knockdown 3255BrM3 cells (data are mean ± SD. Each group contains three mice). **P*<0.05, ***P*<0.01. Scale bar: 10x:50μm.


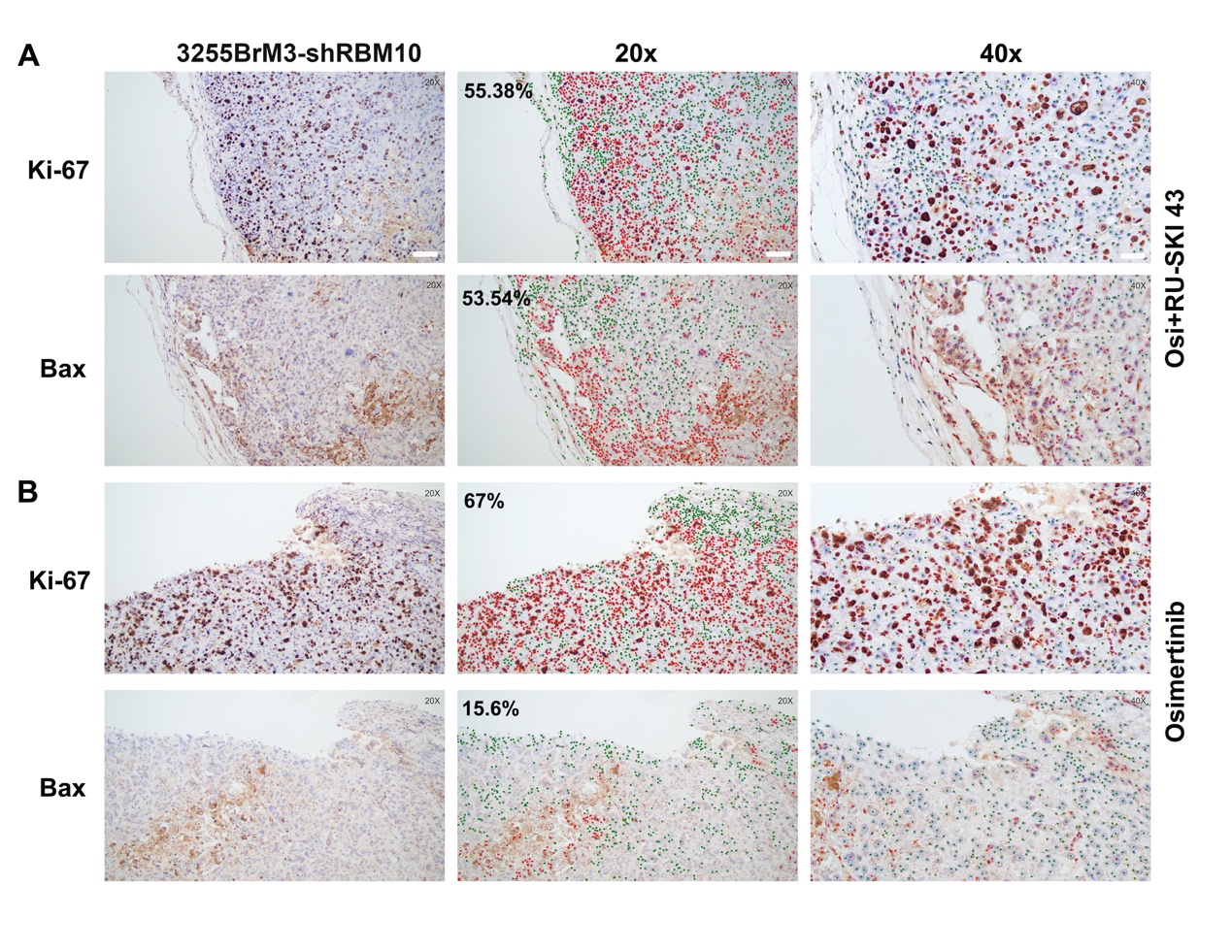


**Figure S10** IHC analysis of Ki-67 and Bax expression in the mice model. (A) In the Osi+RU-SKI43 drug treatment group, the Ki-67 index in 20x tissue sections was 55.38%, and the Bax index was 53.54%. (B) In the Osimertinib drug treatment group, the Ki-67 index in 20x tissue sections was 67%, and the Bax index was 15.6%. Scale bar: 20x:40μm, 40x:20μm.

**Supplementary Tables**

**Table S1 Primary antibody for Western blot and Immunofluorescence**

| Antibody | Manufacturer | Diluted |
| --- | --- | --- |
| GLUT1 | Proteintech,66290-1-Ig | 1:1000 |
| Claudin-5 | Wanleibio, WL03731 | 1:1000/1:200（IF） |
| Zo-1 | Wanleibio, WL03419 | 1:1000/1:200（IF） |
| P-gp | Proteintech,22336-1-AP | 1:1000/1:200（IF） |
| β-actin | ZSGB-BIO, TA-09 | 1:1000 |
| RBM10 | Sigma, HPA034972 | 1:1000 |
| GALC | Proteintech,11991-AP | 1:1000 |
| CK-18 | Proteintech,66187-1-lg | 1:200（IF） |
| GFAP | Proteintech, 23935-1-AP | 1:200（IF） |
| E-cadherin | Proteintech, 20874-1-AP | 1:1000 |
| Vimentin | Proteintech, 10366-1-AP | 1:1000 |
| Ki-67 | Proteintech, 27309-1-AP | 1:2000 |
| Bax | Abmart,T40051F | 1:400 |

IF：Immunofluorescence

**Table S2 Primers sequences for qRT-PCR**

| GENE | Forward (5’to 3’) | Reverse (5’to 3’) |
| --- | --- | --- |
| RBM10 | GCACGACTATAGGCATGACAT | AGTCAAACTTGTCTGCTCCA |
| S1PR1 | TTCCACCGACCCATGTACTAT | GCGAGGAGACTGAACACGG |
| S1PR2 | CATCGTCATCCTCTGTTGCG | GCCTGCCAGTAGATCGGAG |
| S1PR3 | CGGCATCGCTTACAAGGTCAA | GCCACGAACATACTGCCCT |
| S1PR4 | GACGCTGGGTCTACTATTGCC | CCTCCCGTAGGAACCACTG |
| S1PR5 | GCGCACCTGTCCTGTACTC | GTTGGTGAGCGTGTAGATGATG |
| GALC | ACTCTCACCACTGGTCGCAAAG | GATCAGCAAAGTTTGGAGCTTCAC |
| SPHK1 | GCTCTGGTGGTCATGTCTGG | CACAGCAATAGCGTGCAGT |
| SPHK2 | ATGGCATCGTCACGGTCTC | CTCCCAGTCAGGGCGATCTA |
| Ftz | GCAGACGCAGAAGCTGAAGA | GACGCCGGGTGATGTATCTA |
| GAPDH | GACTCATGACCACAGTCCATGC | AGAGGCAGGGATGATGTTCTG |

**Table S3 Frameshift Mutations Identified in RBM10**

| **Exon** | **Transcript ID** | **Mutation** | **Protein Change** |
| --- | --- | --- | --- |
| **13** | NM_001204466 | c.1260delT | p.Ser420fs |
| **13** | NM_152856 | c.1257delT | p.Ser419fs |
| **14** | NM_001204467 | c.1488delT | p.Ser496fs |
| **14** | NM_001204468 | c.1686delT | p.Ser562fs |
| **14** | NM_005676 | c.1491delT | p.Ser497fs |
| **19** | NM_001204466 | c.2096dupG | p.Arg699fs |
| **19** | NM_152856 | c.2093dupG | p.Arg698fs |
| **20** | NM_001204467 | c.2324dupG | p.Arg775fs |
| **20** | NM_001204468 | c.2522dupG | p.Arg841fs |
| **20** | NM_005676 | c.2327dupG | p.Arg776fs |

**Table S4** **Four RBM10 Mutations for Functional Studies**

| **Plasmid ID** | **Exon** | **Transcript ID** | **Mutation** | **Protein Change** |
| --- | --- | --- | --- | --- |
| **E1** | 13 | NM_001204466 | c.1260delT | p.Ser420fs |
| **E2** | 14 | NM_001204467 | c.1488delT | p.Ser496fs |
| **E3** | 19 | NM_001204466 | c.2096dupG | p.Arg699fs |
| **E4** | 20 | NM_001204468 | c.2522dupG | p.Arg841fs |

**Table S5 Primers sequences for minigene splicing reporter assays**

| GENE | Forward (5’to 3’) | Reverse (5’to 3’) |
| --- | --- | --- |
| GALC | CTGAGCCCTCCCACATGCATTAT | GGAGCATGGATGCAGAGATGGAC |

**Table S6 Mutation status and Brain metastasis (BM).**

|  | **BM (-)** | **BM (+)** |
| --- | --- | --- |
| **EGFR Mut** | 16 | 7 |
| **19Del** | 7 | 3 |
| **L858R** | 9 | 2 |
| **L861Q** | 0 | 1 |
| **20-ins** | 0 | 1 |
| **EGFR WT** | 6 | 4 |
| **TP53 Mut** | 0 | 1 |
| **EML4-ALK Fusion** | 2 | 0 |
| **KRAS G12C Mut** | 5 | 1 |
| **ERBB2 Mut** | 1 | 0 |
| **HER-2 20 Mut** | 0 | 1 |
| **BRAF Mut** | 1 | 0 |
| **KRAS G12S/G12D Mut** | 1 | 0 |
| **MET Mut** | 1 | 0 |
